# Radiation image reconstruction and uncertainty quantification using a Gaussian process prior

**DOI:** 10.1038/s41598-024-71336-z

**Published:** 2024-10-03

**Authors:** Jaewon Lee, Tenzing H. Joshi, Mark S. Bandstra, Donald L. Gunter, Brian J. Quiter, Reynold J. Cooper, Kai Vetter

**Affiliations:** 1 Department of Nuclear Engineering, University of California, Berkeley, Berkeley, CA 94720 USA; 2 Nuclear Science Division, Lawrence Berkeley National Laboratory, Berkeley, CA 94720 USA; 3 Gunter Physics, Inc., Lisle, IL 60532 USA

**Keywords:** Imaging techniques, Applied mathematics, Computational science

## Abstract

We propose a complete framework for Bayesian image reconstruction and uncertainty quantification based on a Gaussian process prior (GPP) to overcome limitations of maximum likelihood expectation maximization (ML-EM) image reconstruction algorithm. The prior distribution is constructed with a zero-mean Gaussian process (GP) with a choice of a covariance function, and a link function is used to map the Gaussian process to an image. Unlike many other maximum a posteriori approaches, our method offers highly interpretable hyperparamters that are selected automatically with the empirical Bayes method. Furthermore, the GP covariance function can be modified to incorporate a priori structural priors, enabling multi-modality imaging or contextual data fusion. Lastly, we illustrate that our approach lends itself to Bayesian uncertainty quantification techniques, such as the preconditioned Crank–Nicolson method and the Laplace approximation. The proposed framework is general and can be employed in most radiation image reconstruction problems, and we demonstrate it with simulated free-moving single detector radiation source imaging scenarios. We compare the reconstruction results from GPP and ML-EM, and show that the proposed method can significantly improve the image quality over ML-EM, all the while providing greater understanding of the source distribution via the uncertainty quantification capability. Furthermore, significant improvement of the image quality by incorporating a structural prior is illustrated.

## Introduction

Radiation imaging plays a crucial role in numerous applications including medical diagnostic imaging^1^, non-destructive testing^2^, nuclear contamination remediation, and nuclear safeguards^3^. To this end, various radiation imaging modalities have been proposed, developed, and used in the past, with the goal of imaging the spatial variation of relevant properties such as the radioactivity distribution or attenuation coefficients of intervening materials. However, these properties are difficult to measure directly without physical modulations and subject to statistical noise. As a result, image reconstruction, in which the image of interest is recovered from indirect, noisy measurements, is a crucial part of radiation imaging.

Image reconstruction is often formulated as a statistical inverse problem and conventionally, it is solved with the maximum likelihood estimation (MLE) method^4^. However, the noisy nature of the measurements, combined with various resource constraints in the measurement process often leads to solutions that are unstable and non-unique (i.e., it is an ill-posed inverse problem), adding complexity to the task of accurately interpreting the reconstructed images. Hence, an image reconstruction technique capable of improving the MLE solution and estimating uncertainties in the reconstructed images is of significant value.

Bayesian image reconstruction, in which a priori information is incorporated into an image reconstruction problem, is an attractive alternative to the MLE approach^5^. In the Bayesian paradigm, image reconstruction is often posed as a maximum a posteriori (MAP) problem, where the image is recovered by locating the mode of the posterior distribution. Various MAP techniques have been studied extensively in the context of image reconstruction, ranging from Tikhonov regularization^6^, total variation (TV)^7^ regularization to plug-and-play priors^8^ that utilize pre-trained deep neural networks. It has been shown that the MAP approaches can significantly improve the quality of reconstructed images compared to the MLE, while alleviating the ill-posedness of the image reconstruction problem^9^. Moreover, unlike frequentist approaches such as MLE, the Bayesian paradigm offers a straightforward interpretation of uncertainties, including both aleatoric uncertainties, arising from the inherent randomness in data, and epistemic uncertainties, resulting from a lack of data^10^.

However, the Bayesian image reconstruction paradigm often faces several challenges. First, the hyperparameters (i.e., regularization parameters) reflecting a priori knowledge are often difficult to choose in a principled manner^11^. Secondly, even with carefully selected hyperparameters, the prior model itself may not be flexible enough to model the a priori knowledge that needs to be incorporated. Lastly, Bayesian uncertainty quantification of reconstructed images require very high dimensional integration, and analytical solutions are generally unavailable unless conjugate priors are employed^12^.

To tackle the aformentioned challenges, we propose the use of a Gaussian process (GP)-based prior for image reconstruction and uncertainty quantification tasks. A GP is a stochastic process where the constituent random variables jointly follow a multivariate Gaussian distribution^13^. The GPs can be made very flexible with a choice of mean and covariance functions, and they have been extensively studied in the context of regression tasks^14^. In this work, we demonstrate that GPs can also serve as a powerful prior for Bayesian image reconstruction tasks. The proposed approach to use a GP prior will be referred to as Gaussian process prior (GPP) henceforth. The following are the contributions of our work:

- First, we demonstrate that the MAP estimate with the proposed GPP can significantly enhance the quality of the reconstructed radiation images compared to the conventional MLE-based approach. The computational cost in using the GP is greatly eased by exploiting the Kronecker product structure in the GP covariance matrix.
- We then show that uncertainties in the reconstructed images can be quantified using the preconditioned Crank–Nicolson (pCN) algorithm^15^ and the Laplace approximation^16^^17^.
- We illustrate that the flexibility of the GPP allows for incorporation of structural prior, which can dramatically improve the reconstruction in some imaging scenarios.
- Lastly, we lay out a principled method for choosing the hyperparameters of the GPP, using the empirical Bayes method and Laplace approximation.

We note that a Bayesian approach using a GP prior for image reconstruction and uncertainty quantification was recently introduced by Zhou et al.^18^, where a combination of a TV and a GP prior and the pCN-based uncertainty quantification method were proposed. However, due to the non-differentiable nature of the TV prior, it limits the use of gradient-based optimization and Hessian information. In contrast, in this work, we demonstrate that the Laplace approximation, which leverages the Hessian of the posterior, enables scalable, yet effective uncertainty quantification and hyperparameter selection.

The proposed framework is general and can be used for most radiation imaging modalities with Poisson likelihood and (with a small modification) Gaussian likelihood. In this work, we show the effectiveness of the proposed method with free-moving single detector imaging scenarios, which is based on the recently developed scene data fusion (SDF) concept^3,19,20^. We chose this imaging modality for two reasons: firstly, due to the insufficient angular modulation from a single detector and vast imaging space, the imaging problem is highly ill-posed. As a result, the solutions found from the ML-EM algorithm are often undesirable^21^, highlighting the benefits of the GPP image reconstruction and uncertainty quantification methods. Secondly, there has been growing interest in the free-moving imaging modalities due to its significant impact in nuclear safety and security applications^22^; however, the concept is often challenged by the absence of reliable image reconstruction methods. Hence the proposed approach can provide accurate reconstruction of radiation fields for a wide range of applications, thereby addressing the current needs of the research field.

## Results

In this section, we demonstrate the effectiveness of the proposed GPP MAP image reconstruction and uncertainty quantification techniques using simulated free-moving single detector imaging scenarios.

Each free-moving measurement scenario was simulated with a 10-cm diameter spherical detector (i.e., isotropic response) in a 20 m × 20 m 2-D imaging space. The detector intrinsic efficiency was assumed to be 10% and the detector moving paths were simulated on the x-y plane. In all measurement scenarios, the z-coordinates of the pixel centers and detector paths were fixed at 0 m and 0.5 m, respectively. Along the detector paths, recorded counts were binned with the integration time of 0.1 s.

### Anisotropic Gaussian Source

**Fig. 1:**
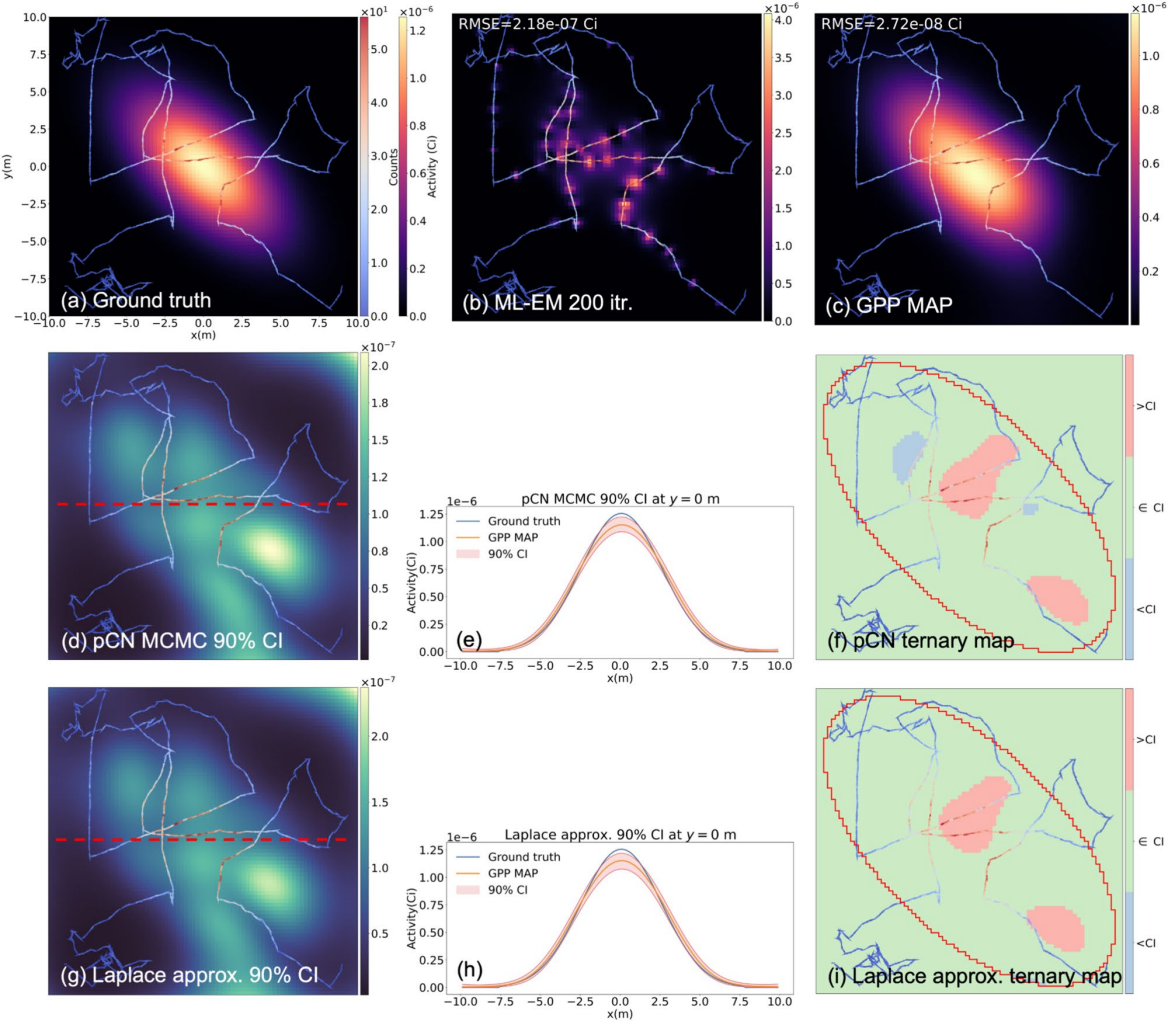
(**a**) A simulated measurement scenario with a 1-mCi anisotropic Gaussian distributed source. The human-walked path is marked with arrows and colored according to the measured counts. (**b**) The reconstructed image from the ML-EM algorithm after 200 iterations. (**c**) The reconstructed from the proposed GPP MAP. The hyperparameters $\sigma$=4.1 m and $\lambda =1.4\,\times \,10^{-4}$ $\text {Bq}^{-1}$ found from the empirical Bayes method were used for reconstruction. (**d**,** g**) The pixelwise 90% credible interval maps from the pCN and the Laplace approximation, respectively. (**e**,** h**) The credible intervals and the ground truth at *y*=0 m (red dotted lines in (**d**) and (**g**)). (**f**,** i**) Ternary maps in which the pixels are colored differently based on the ground truth pixel values relative to the estimated 90% credible intervals.

First, a measurement scenario with an anisotropic Gaussian source was simulated to demonstrate the effectiveness of the proposed algorithm. Gaussian source distributions are of particular interest because radioactive material dispersion often exhibits Gaussian-like distributional features^23^.

Fig. 1a illustrates the simulated 1-mCi anisotropic Gaussian distributed source positioned at the center of the imaging space and the 150-m path of the moving detector. Assuming the average human walking speed of 1.5 m/s, the total measurement time amounted to 98 s.

Fig. 1b,c show the image reconstruction results of the ML-EM after 200 iterations and the proposed GPP algorithm. As can be evidently seen, the ML-EM reconstructed image exhibits high frequency features clustered around the path, and we observed such patterns even after 20 iterations. On the other hand, the image reconstructed with the GPP MAP successfully localizes and maps the Gaussian distribution.

**Fig. 2:**
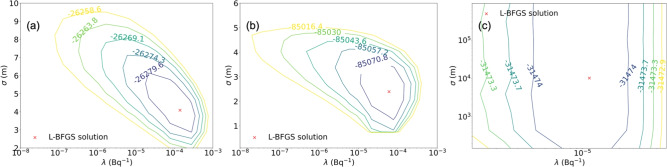
Contour plots of the negative log-marginal likelihood as a function of the hyperparameters $\sigma$ and $\lambda$. The hyperparameters selected from Algorithm 2 are marked with red Xs. (**a**) The anisotropic Gaussian source measurement scenario. (**b**) The ring and Gaussian source measurement scenario. (**c**) The uniform square source measurement scenario with a structural prior. When using the structural prior, 2 different sets of hyperparameters were used for in-square and out-of-square. Therefore, only the in-square hyperparameters were used to create the contour plot, while the out-of-square hyperparameters were fixed at the values found from Algorithm 2.

**Fig. 3:**
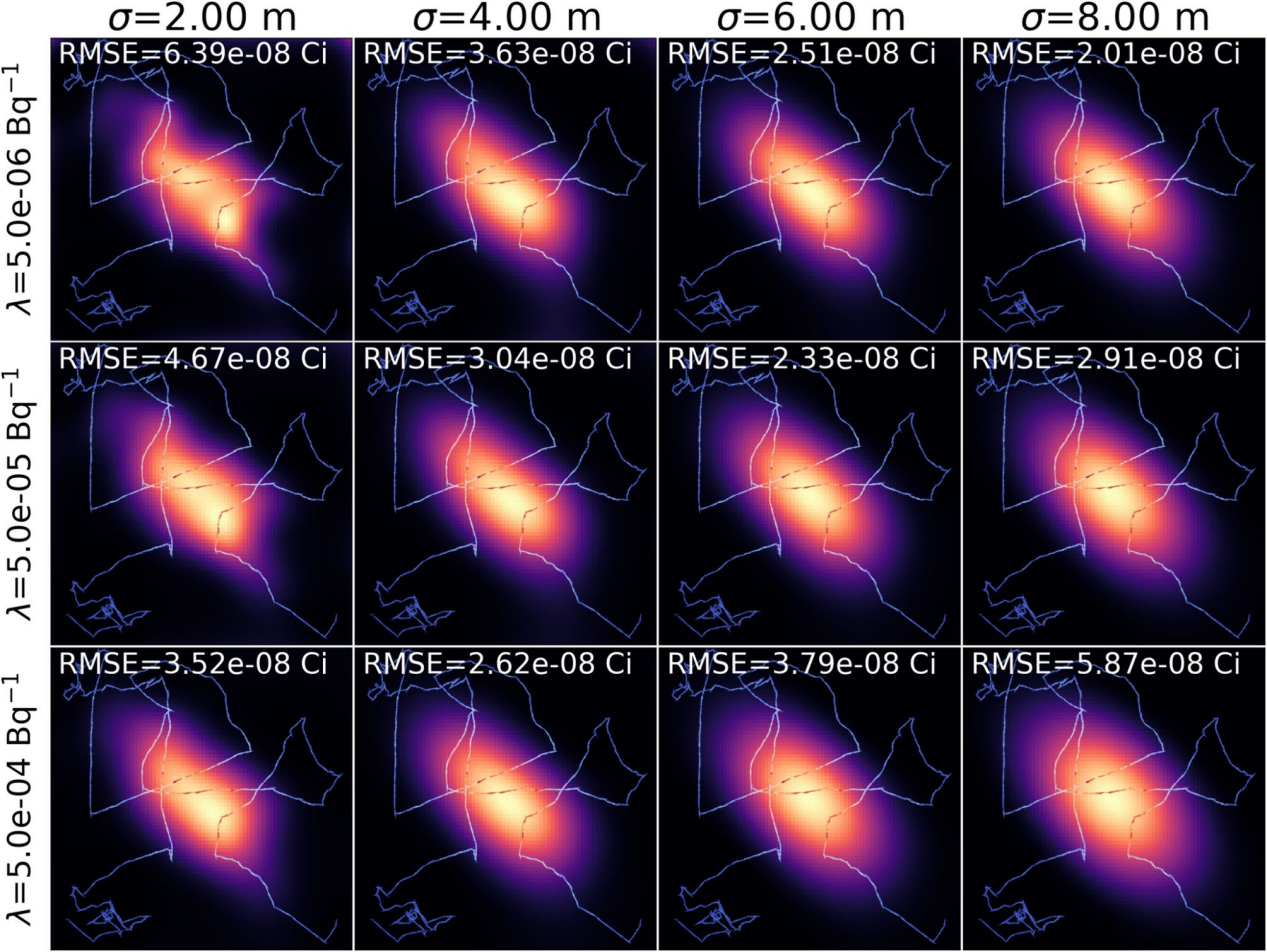
To better understand the impact of the hyperparameters, the anisotropic Gaussian source images reconstructed from the GPP MAP with different choices of the hyperparameters $\sigma$ and $\lambda$ are displayed. With larger $\sigma$ and $\lambda$, the prior distribution is spatially more correlated and the pixel values are pushed towards zeros. As a result, the reconstructed image with large hyperparameters (bottom right) tend to spread out.

The hyperparameters of the GPP algorithm ($\sigma =\text {4.1 m}$ and $\lambda =1.4\times 10^{-4}$ $\text {Bq}^{-1}$) were chosen with Algorithm 2. Fig. 2a shows the contour plot of the negative log-marginal likelihood as a function of $\sigma$ and $\lambda$, together with the optimal hyperparameters selected from Algorithm 2. To investigate the impact of hyperparameter choices on image reconstruction, Fig. 3 shows the GPP MAP reconstructed images with various hyperparameters. As illustrated, the algorithm performs well over a large range of hyperparameter choices.

Fig. 1d,g show the pixel-wise 90% credible interval maps from the pCN and Laplace approximation, respectively. For the pCN MCMC, $10^6$ samples were collected and the pixel-wise credible intervals were computed. In both cases, there exists about 10% of uncertainty around the source region, due to the ambiguity in the reconstructed images. Interestingly, the uncertainty is lower in those pixels close to the path, reflecting the fact that the source activity can be inferred with more confidence in the pixels with large sensitivity. Conversely, the unexplored region in the upper right corner exhibits high uncertainties, because the measurement is not sensitive to the pixels in the region. Note that with the Laplace approximation, the uncertainties in the upper right corner are slightly overestimated. We suspect that the marginal pixel posteriors in the upper right corner are exponentially distributed due to the lack of measurements, (i.e., prior $\sim$ posterior). Capturing the skewness of the exponential distribution is challenging with the Laplace approximation because of the Gaussian posterior assumption. However, from Fig. 1e,h, which depict the slices of the credible interval maps at $y=0$ m, it can be observed that both uncertainty quantification methods produce very similar results at the center of the imaging space where the source is distributed.

Fig. 1f,i display the ternary maps, where 3 different colors are used depending on the ground truth pixel values relative to the estimated credible intervals. The pixels where the ground truth values are bounded by the credible intervals are colored green, while red and blue indicate the ground truth values exceeding and falling below the credible intervals, respectively. As illustrated, in most pixels the ground truth is well bounded by the credible intervals estimated from both the pCN and Laplace approximation.

### Ring and Gaussian source

**Fig. 4:**
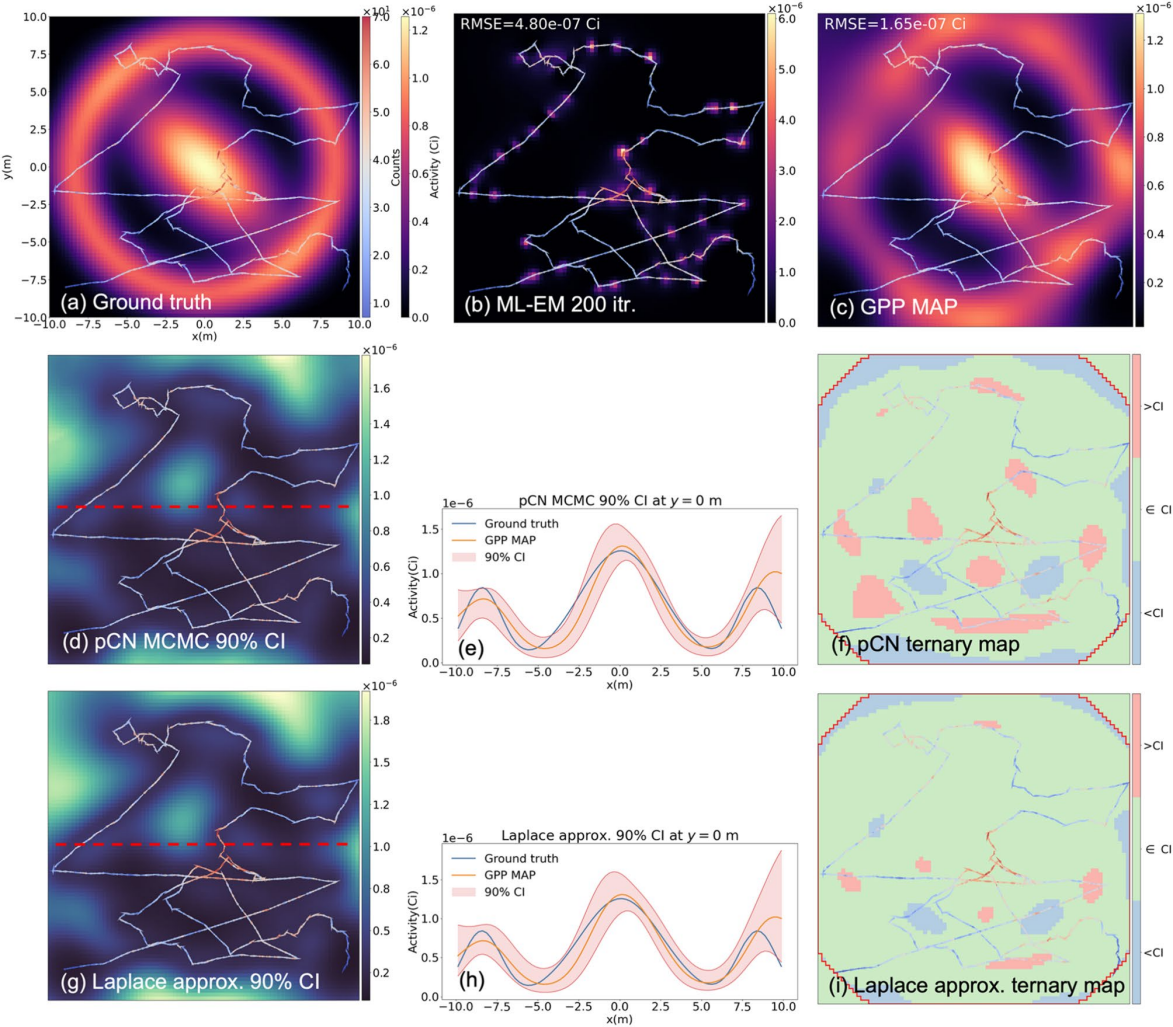
(**a**) A simulated measurement scenario with a 2-mCi ring-shaped source and a 1-mCi anisotropic Gaussian distributed source. The detector moving path is marked with arrows and colored according to the measured counts. (**b**) The reconstructed image from the ML-EM algorithm after 200 iterations. (**c**) The reconstructed from the proposed GPP MAP. The hyperparameters $\sigma$=2.4 m and $\lambda =6.0\,\times \,10^{-5}$ $\text {Bq}^{-1}$ found from the empirical Bayes method were used for reconstruction. (**d**,** g**) The pixelwise 90% credible interval maps from the pCN and the Laplace approximation, respectively. (**e**, **h**) The credible intervals and the ground truth at *y*=0 m (red dotted lines in (**d**) and (**g**)). (**f**, ** i**) Ternary maps in which the pixels are colored differently based on the ground truth pixel values relative to the estimated 90% credible intervals.

A more challenging measurement scenario involving a 2 mCi ring-shaped source and a 1 mCi anisotropic Gaussian source was simulated as depicted in Fig. 4a along with the detector moving path. The total measurement duration was approximately 100 s. As can be seen in Fig. 4b,c, while the ML-EM produces source distribution concentrated around the measurement path, the GPP MAP estimate offers significantly more improved reconstruction results. The GPP hyperparameters were set to $\sigma =\text {2.4 m}$ and $\lambda =\text {6.0}\times \text {10}^{-5}\text { Bq}^{-1}$, which were selected with Algorithm 2. The contour plot of the negative marginal log-likelihood as a function of the hyperparameters can be found in Fig. 2b.

Fig. 4d,g present the results of the uncertainty quantification using the pCN and Laplace approximation, respectively. Similarly to the findings in Sect. 2.1, the pixel uncertainties are predominantly influenced by the sensitivities of the pixels; uncertainties are lower around the detector path and elevated in those unvisited regions. Also, the Laplace approximation slightly overestimates the uncertainties, yet overall it remains largely comparable to the pCN as can be seen in Fig. 4e,h. In addition, the ternary maps Fig. 4f,i demonstrate that the ground truths are mostly bounded by the credible intervals estimated from the both methods.

**Figure 5:**
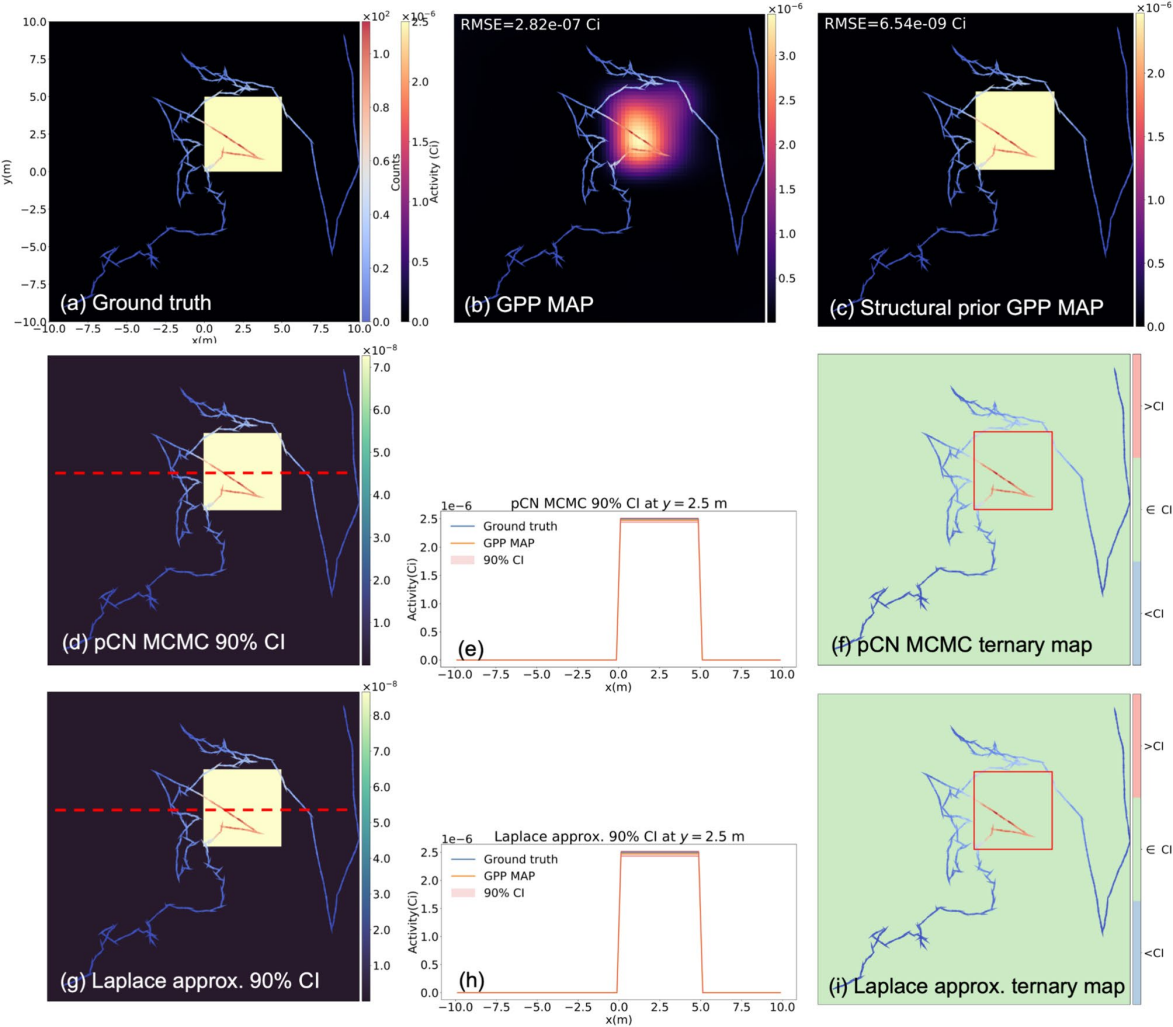
(**a**) A simulated measurement scenario with a 1-mCi uniform located at $(x,y)=$  (2.5m, 2.5m). The detector moving path is marked with arrows and colored according to the measured counts. (**b**) The reconstructed image from the GPP MAP without incorporating the structural information. The hyperparameters found from Algorithm 2, $\sigma$ = 2.03 m and $\lambda$ = 8.10 Bq$^{-1}$ were used. (**c**) The reconstructed from the proposed GPP MAP with the structural prior. $\sigma =1.0\times 10^4$ m and $\lambda =1.1\times 10^{-5}$ Bq$^{-1}$ for the in-square hyperparameters, and $\sigma =9.8\times 10^3$ m and $\lambda =7.7\times 10^4$ Bq$^{-1}$ for the out-of-square hyperparameters. (**d**,** g**) The pixelwise 90% credible interval maps from the pCN and the Laplace approximation, respectively. (**e**,** h**) The credible intervals and the ground truth at *y* = 2.5 m (red dotted lines in (**d**) and (**g**)). (**f**,** i**) Ternary maps in which the pixels are colored differently based on the ground truth pixel values relative to the estimated 90% credible intervals.

### Uniform square source with a structural prior

We also demonstrate that incorporation of a structural prior using the GPP algorithm could significantly improve the image quality. Fig 5a show another measurement scenario with a 1 mCi uniform square source locate at (*x*, *y*) = (2.5 m, 2.5 m). The total measurement duration was approximately 70 s. The reconstruction result using the GPP MAP is shown in Fig. 5b. Although the GPP MAP reconstruction successfully localized the source, the high frequency edges could not be recovered for two reasons: first, due to the lack of modulations in the single detector measurements, the forward projected measurements are blurred and noisy, leading to some ambiguity in the reconstructed images. Second, the GP prior with the SE kernel is not apt to model the high frequency edges of the ground truth distribution.

Here we show that the limitations can be overcome by incorporating a priori structural information. The prior covariances between the pixels within and outside the source square can be zeroed to enforce independence between the two pixel clusters. The reconstruction result with the structural prior is presented in Fig. 5c. As can be observed, the reconstruction successfully recovers the uniform ground truth distribution with the edges. Note that two different sets of hyperparameters were used for the two pixel clusters, namely the in-square hyperparameters and the out-of-square hyperparameters. Using Algorithm 2, the hyperparameters were set at $\sigma =1.0\times 10^4$ m and $\lambda =1.1\times 10^{-5}$ $\text {Bq}^{-1}$ for the in-square hyperparameters, and $\sigma =9.8\times 10^3$ m and $\lambda =7.7\times 10^4$ $\text {Bq}^{-1}$ for the out-of-square hyperparameters. The very large $\sigma$ hyperparameters for both pixel clusters indicate that a piecewise constant function with the discontinuities at the pixel cluster boundaries well-explain the measurement data. Also the large difference in the $\lambda$ parameters between the pixel clusters reflects the fact that the source is only present in the in-square pixel clusters. Fig. 2c shows a contour plot illustrating the negative log-marginal likelihood as a function of in-square hyperparameters.

The 90% credible interval maps found from the pCN and Laplace approximation are displayed in Fig. 5d,g, respectively. Note that due to the strong correlations between the pixel clusters, the marginal credible interval maps are also step-function like. Also, similarly to the findings from the results presented in Sects. 2.1 and 2.2, a slight overestimation of uncertainties was observed with the Laplace approximation. However, both methods produce comparable results as can be observed from Fig. 5e,h, where the slices of the uncertainty maps at $y=\text {2.5 m}$ are shown. Also, the ternary maps in Fig. 5f,i indicate that the ground truth distribution is well-bounded by the estimated uncertainties.

## Discussion

In this section, we discuss the implications of the proposed methods and potential future research directions.

### Implications of the proposed approach

First, the proposed GP prior can be made highly flexible with a choice of a covariance function and a link function. Therefore, the GP prior can effectively model desired properties of the reconstructed images, and is directly applicable to a wide range of highly ill-posed image reconstruction tasks. Such tasks arise from various radiation imaging modalities including Compton cameras^24^, coded aperture imaging^25^, limited angle computed tomography (CT)^26^, low-dose positron emission tomography (PET)^27^, among others.

Secondly, the uncertainty quantification capability of the proposed framework offers valuable insights into the reconstructed images, and it becomes particularly important when critical decisions are made based on the reconstructed images. Such problems commonly arise in many healthcare^28^ and nuclear security applications^29^. Moreover, posterior distributions estimated from the pCN and the Laplace approximation can be employed for further inferences on quantities derived from the reconstructed images. For instance, suppose a quantity of interest *u* can be derived from an image $\textbf{x}$ as $u=g(\textbf{x})$. From a set of posterior samples obtained from the pCN, $\{\varvec{\xi }_n\}_{n=1}^J$, the samples of the derived quantity $\{u_n\}_{n=1}^J$ can be obtained as $u_n = g(\varvec{f}^{-1}(\varvec{\xi }_n))$. In the context of the Laplace approximation, samples of $\varvec{\xi }$ can be obtained from the approximating Gaussian distribution in (29). Some examples of such derived quantities are total dose registered in radiopharmaceutical therapy^30^, in-beam ion ranges in ion cancer therapy, and in-vivo proton range verification in ion cancer therapy^31^.

### Future works

#### Incorporation of background contribution

The results presented in section demonstrate that the proposed GPP algorithm can significantly improve the reconstruction quality where the ML-EM may be inadequate. However, the simulated measurement scenarios assume no present background contributions, which may be unrealistic in some radiation imaging applications. When the background contribution is known, it can be straightforwardly accounted for by changing the forward projection $\bar{\textbf{y}}=\textbf{A}\textbf{x}$ to $\bar{\textbf{y}}=\textbf{A}\textbf{x} + \textbf{b}$, where the elements of the vector $\textbf{b}$ are the known background contribution to the measurements. When the background rate is unknown, it has to be jointly estimated with the image $\textbf{x}$, and it can be done by augmenting the system matrix $\textbf{A}$ and the image vector $\textbf{x}$ as $\tilde{\textbf{A}} = [\textbf{A}|\textbf{a}_b]\quad \text {and} \quad \tilde{\textbf{x}} = \left[ \frac{\textbf{x}}{b}\right]$, where the vector $\textbf{a}_b$ is assumed to be known (e.g., in the case of a constant background rate, a vector of integration time $\textbf{a}_b$ = $\Delta t \mathbb {1}$). Subsequently, the prior covariance matrix $\Sigma$ can be augmented as

1 $$\begin{aligned} \tilde{\varvec{\Sigma }} = \begin{bmatrix} \varvec{\Sigma } & \varvec{0}\\ \varvec{0} & \Sigma _b \end{bmatrix}, \end{aligned}$$

where the $\Sigma _b$ accounts for the variance of the background rate.

The use of the Gaussian-to-exponential link function may be inadequate as the background rate can be typically well specified within an order of magnitude. In such case, a different link function, such as the truncated Gaussian-to-Gaussian link function^32^, can be used to effectively model the background prior distribution.

#### List-mode data and Gaussian likelihood

Although the work presented here assumes bin-mode data with the Poisson likelihood model, it should be emphasized that the proposed framework can be trivially modified to accommodate list-mode data^33^ or Gaussian likelihood model. Essentially, the only differences in the formulation will be the likelihood function formulation in (4), and subsequently its gradient, Hessian expressions.

For example, when the measurements are given in terms of dose rates, Gaussian likelihood model can more aptly describe the measurement noise. In such cases, the likelihood function $l(\textbf{x};y)$ in (5) can be modified as follows:

2 $$\begin{aligned} l(\textbf{x};y) = \frac{1}{2}(\textbf{y} - \textbf{Ax})^{\textsf{T}} \textbf{W} (\textbf{y} - \textbf{Ax}), \end{aligned}$$

where the matrix $\textbf{W} \in \mathbb {R}^{M\times M}$ is the inverse of the measurement covariance matrix (i.e. a precision matrix). When independent and identical noise distribution is assumed, $\textbf{W}$ is an identity matrix. With the Gaussian likelihood model, the GPP image reconstruction and uncertainty quantification presented in this paper can be similarly applied.

By accommodating various data types, the proposed framework is applicable to a wide range of radiation imaging applications.

#### Scalable implementation

In Sect. 4.1, we illustrated how the Kronecker product structure in the covariance matrix $\varvec{\Sigma }$ can be exploited to avoid the computational challenges involving the Cholesky factor $\textbf{L}$. The approach is particularly attractive as it provides exact representation of the covariance matrix; however, the method is not readily applicable when the imaging space is not in a dense Cartesian grid. For example, Compton cameras or active coded apertures imagers often employ a 4$\pi$ spherical imaging space^34,35^, and free-moving imaging scenarios often assume sparse voxel grid imaging spaces^36^. Thus, a different strategy is needed when more diverse imaging spaces are considered.

Several recent advances from the GP regression research community could potentially be employed to tackle the challenges. For example, in^37^, Willson et al. introduced the structured kernel interpolation (SKI) scheme, where the covariance matrix is interpolated from a Kronecker product represented sparse inducing points. The resulting approximate covariance matrix can be handled efficiently thanks to the sparsity of the interpolation coefficient matrix and the Kronecker product structure. Such formulation could be applied to imaging spaces on a non-structured grid.

Another interesting approach is Gaussian Markov random field (GMRF), where a sparse precision matrix $\varvec{\Sigma }^{-1}$ is directly constructed and utilized. Such precision matrices admit rapid realization of GPs, removing the computational bottlenecks involving $\textbf{L}\varvec{\xi }_w$. Moreover, Lindgren et al. showed that a GMRF can directly approximate GPs with a Matérn covariance kernel, and it can be efficiently applied to meshed imaging spaces^38^.

#### Covariance Kernels

The SE kernel in (11) is a popular choice in many GP regression tasks, and in this work we demonstrated that it can be used to effectively model the prior covariance. In many radiation imaging modalities, the point spread functions (PSF) have smoothly falling edges and therefore, the use of the SE kernel could be well-justified. However, when it is required to recover high frequency features (e.g., edges or point sources), more expressive kernels may be desired. Rational quadratic kernel or Matérn covariance kernels could be readily used to provide more flexibility for better high frequency feature resolving capability. Moreover, recent works from the GP regression community suggest the use of spectral mixture (SM) kernel^39^ or deep kernel learning framework^40^, where a deep neural network is used to construct a highly expressive covariance kernel. It was demonstrated in^40^ that such kernels can successfully recover piecewise constant functions with sharp falling edges, which is attractive for imaging applications.

## Methods

### Image reconstruction with GPP

#### GPP MAP image reconstruction

The goal of radiation imaging is to recover an *N*-dimensional image vector $\textbf{x} \in \mathbb {R}_{\ge 0}^N$ from an *M*-dimensional non-negative measurement vector $\textbf{y} \in \mathbb {Z}_{\ge 0}^M$. The two vectors are related by a system matrix $\textbf{A}\in \mathbb {R}_{\ge 0}^{M \times N}$ such that

3 $$\begin{aligned}&\textbf{y} \sim {\text {Poisson}}(\bar{\textbf{y}}),\quad \text {where}\quad \bar{\textbf{y}} = \textbf{Ax}. \end{aligned}$$

Hence the system matrix contains the probabilities of radiation emitted from a voxel being detected in a measurement bin. With the Poisson measurement model, the likelihood function of an image vector $\textbf{x}$ given the measurement vector $\textbf{y}$ can be formulated as

4 $$\begin{aligned} p(\textbf{y}|\textbf{x}) = \prod _{i=1}^M \frac{e^{-\bar{y}_i}\bar{y}_i^{y_i}}{y_i!}. \end{aligned}$$

The well-established maximum likelihood (ML) image reconstruction approach seeks a solution $\hat{\textbf{x}}_{\text {ML}}$, which minimizes the negative log-likelihood function $l(\textbf{x}{;}\textbf{y}) =-\log {p(\textbf{y}|\textbf{x})}$ up to a constant as follows:

5 $$\begin{aligned} \hat{\textbf{x}}_{\text {ML}}&= \mathop {\text{argmin}}\limits _{\textbf{x}, \textbf{x} \succeq 0} \, l(\textbf{x};\textbf{y}) \end{aligned}$$

6 $$\mathop = \limits^{c} \mathop {{\text{argmin}}}\limits_{{{\mathbf{x}},{\mathbf{x}}{ \succcurlyeq }0}} \sum\limits_{{i = 1}}^{M} {\bar{y}_{i} } - y_{i} \log \bar{y}_{i}$$

7 $$= \mathop {{\text{argmin}}}\limits_{{{\mathbf{x}},{\mathbf{x}}{ \succcurlyeq }0}} \sum\limits_{{i = 1}}^{M} {\left[ {{\mathbf{Ax}}} \right]_{i} } - y_{i} \log \left[ {{\mathbf{Ax}}} \right]_{i} ,{\text{ }}$$

where $\textbf{x}\succeq 0$ imposes the non-negativity constraints and ${\mathop {=}\limits ^{c}}$ denotes equivalence up to a constant. It is well-known that the above optimization problem can be iteratively solved using the expectation maximization (EM) algorithm, leading to the popular maximum likelihood-expectation maximization (ML-EM) algorithm^41^.

Although ML-EM has become a standard approach for image reconstruction tasks, for many radiation imaging problems the system matrix $\textbf{A}$ is often not well-conditioned. Hence, the optimization problem (5) is ill-posed and the ML-EM algorithm is susceptible to overfitting.

A popular approach to combat the ill-posedness is to incorporate a prior distribution $p(\textbf{x})$ to make use of Bayesian formalism. Using Bayes’ rule^42^, the posterior distribution $p(\textbf{x}|\textbf{y})$ is given by

8 $$\begin{aligned} p(\textbf{x}|\textbf{y}) = \frac{p(\textbf{y}|\textbf{x})p(\textbf{x})}{p(\textbf{y})}. \end{aligned}$$

In Bayesian image reconstruction, often the image vector is recovered by locating the mode of the posterior distribution, leading to the Maximum a Posteriori (MAP) estimation as follows:

9 $$\begin{aligned} \hat{\textbf{x}}&= \mathop {\text{argmin}}\limits _{\textbf{x}, \textbf{x} \succeq 0} \, l(\textbf{x};\textbf{y}) + l_p(\textbf{x}), \end{aligned}$$

where $l_p(\textbf{x}) = -\log {p(\textbf{x})}$ is the negative log prior distribution. An appropriate choice of a prior distribution is crucial for reliable image reconstruction, especially when the system matrix $\textbf{A}$ is ill-conditioned.

In this work, for prior model construction, we assume that the image vector $\textbf{x}$ is governed by a latent variable $\xi$, which follows a zero-mean Gaussian process (GP),

10 $$\begin{aligned} \xi \sim \mathscr{G}\mathscr{P}(\textbf{0}, k(\textbf{r}, \textbf{r}^{\prime })), \end{aligned}$$

where $k(\textbf{r}, \textbf{r}')$ is a covariance kernel, and $\textbf{r}, \textbf{r}^{\prime } \in \mathbb {R}^3$ represents vectors containing the Cartesian coordinates of two points in the imaging space.

The choice of a covariance kernel $k(\textbf{r}, \textbf{r}^{\prime })$ is crucial as it dictates the underlying spatial correlation of the image. In this work, we use the unit-variance squared exponential (SE) kernel to encode spatial correlation between pixels as follows:

11 $$\begin{aligned} k(\textbf{r}, \textbf{r}^{\prime }) = \exp {\left( -\frac{\left\Vert \textbf{r}-\textbf{r}^{\prime } \right\Vert ^2}{{2\sigma ^2}}\right) }, \end{aligned}$$

where $\sigma$ is the characteristic correlation length scale parameter of the prior distribution with the dimension of distance. It should be noted that various covariance kernels have been explored in the context of GPs, and the optimal covariance kernel may vary depending on applications^43^.

For practical purposes, the imaging space is discretized with pixels and the GP in (10) is evaluated at the pixel centers. Then, the GP reduces to a zero-mean multivariate Gaussian distribution,

12 $$\begin{aligned}&\varvec{\xi } \sim \mathscr {N}(\textbf{0}, \varvec{\Sigma }) \end{aligned}$$

13 $$\begin{aligned}&\text {where} \quad \Sigma _{ij} = k(\textbf{r}_i, \textbf{r}_j). \end{aligned}$$

Hence,

14 $$\begin{aligned} p(\varvec{\xi }) = \frac{1}{\sqrt{(2\pi )^{n}|\varvec{\Sigma }|}}\exp {\left( -\frac{1}{2}\varvec{\xi }^{\textsf{T}}\varvec{\Sigma }^{-1}\varvec{\xi }\right) }. \end{aligned}$$

**Fig. 6:**
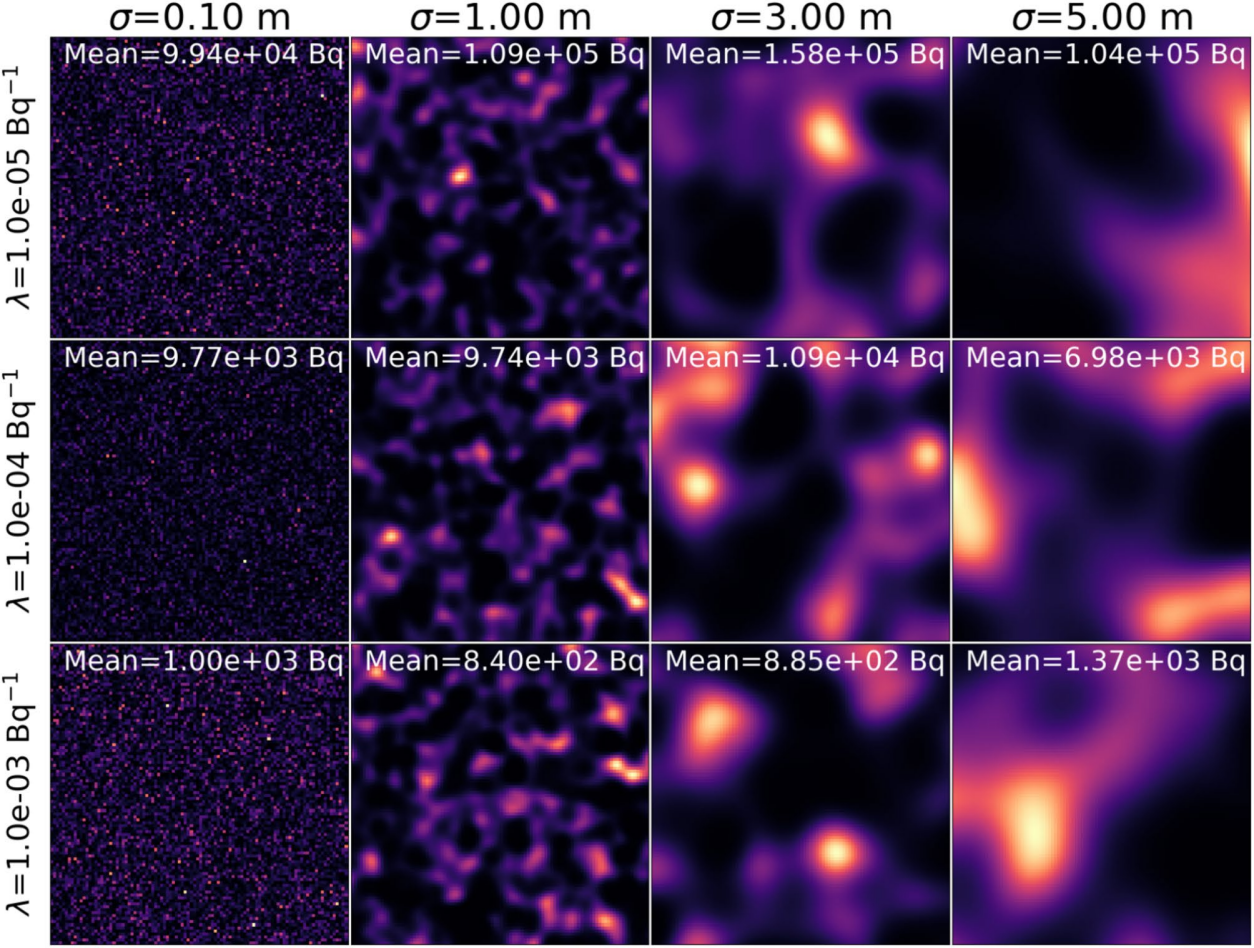
Random samples from the prior distribution $p(\textbf{x})$ on a 20 × 20 m 2-D imaging space with different sets of hyperparameters, $\sigma$ and $\lambda$. Each sample was realized by first drawing a sample from $\varvec{\xi }_w \sim \mathscr {N}(\textbf{0}, \textbf{I})$ and then transforming it by $\textbf{x} = \textbf{f}^{-1}(\textbf{L}\varvec{\xi }_w)$. The properties and characteristics of the prior distribution can be better understood by examining random samples from the prior. Note that the $\sigma$ parameter defines the characteristic correlation length, while the reciprocal of $\lambda$ parameter determines the mean pixel values.

Once the prior $p(\varvec{\xi })$ is defined, a link function $f : \mathbb {R}_{\ge 0}^N \longrightarrow \mathbb {R}^N$ is used to relate $\varvec{\xi }$ and $\textbf{x}$, keeping the positivity constraints imposed on $\textbf{x}$. There are a variety of choices for the link function; for example, a simple logarithmic function, $f(x_i) = \log {x_i} = \xi _i$ could be used as a link function satisfying the non-negativity constraint on $\textbf{x}$. However, for simpler statistical interpretation, we use the exponential-to-Gaussian link function^32^, and its inverse can be used to recover $\textbf{x}$ as follows:

15 $$\begin{aligned} \textbf{x}&= \varvec{f}^{-1}(\varvec{\xi }) =-\frac{1}{\lambda }\log {\left( \frac{1}{2}-\frac{1}{2}{\text {erf}}\left( {\varvec{\xi }}\oslash {(\sqrt{2}{\text {diag}}{(\varvec{\Sigma })})}\right) \right) }, \end{aligned}$$

where $\lambda$ is the scale parameter for the exponential distribution and ${\text {erf}}$ is the error function. $\oslash$ denotes the element-wise division operator. The link function converts a zero-mean Gaussian distributed random variable $\xi _i$ with the variance $\Sigma _{ii}$ into a random variable following the exponential distribution with the scale parameter $\lambda$. As the reciprocal of $\lambda$ defines the mean value of the exponential distributed random variable, it can be used to incorporate *a priori* information about the average pixel value, allowing for more statistically interpretable hyperparameter selection. Furthermore, due to the long-tailed nature of the exponential distribution, the link function can effectively model radiation source strength that varies over several orders of magnitudes. Samples from the prior distribution $p(\textbf{x})$ with different hyperparameters $\sigma$ and $\lambda$ are shown in Fig. 6.

It should be emphasized that the appropriate choice of a link function may vary depending on the application, and the methods presented in this work are applicable with various link functions.

From (14), the negative log-prior for $\varvec{\xi }$ can be written as follows:

16 $$\begin{aligned} l_p(\varvec{\xi })&= -\log {p(\varvec{\xi })}\nonumber \\&= \frac{1}{2}\log {\sqrt{(2\pi )^n|\varvec{\Sigma }|}} + \frac{1}{2}\varvec{\xi }^{\textsf{T}}\varvec{\Sigma }^{-1}\varvec{\xi } \end{aligned}$$

Combining (15) and (16), the MAP solution in terms of ${\varvec{\xi }}$ can be obtained by minimizing the unnormalized negative log posterior $\Psi (\varvec{\xi }) {\mathop {=}\limits ^{c}} l(\varvec{\xi };\textbf{y}) + l_p(\varvec{\xi })$ as follows:

17 $$\begin{aligned} \hat{\varvec{\xi }}&= \mathop {\text{argmin}}\limits _{{\varvec{\xi }}} \, \Psi (\varvec{\xi }) \end{aligned}$$

18 $$= \mathop {\text{argmin}}\limits _{{\varvec{\xi }}} \sum _i^N [\textbf{A}\varvec{f}^{-1}(\varvec{\xi }) -\textbf{y}\odot \log {(\textbf{A}\varvec{f}^{-1}(\varvec{\xi }))}]_i + \frac{1}{2}\varvec{\xi }^\top \varvec{\Sigma }^{-1}\varvec{\xi }.$$

The optimization problem (18) can be solved in a more efficient and numerically stable manner by introducing an uncorrelated multivariate unit Gaussian variable $\varvec{\xi }_w \sim \mathscr {N}(\varvec{0}, \textbf{I})$. Hence, we use a change of variable $\textbf{L}\varvec{\xi }_w = \varvec{\xi }$, where $\textbf{L}$ is the Cholesky factor of $\varvec{\Sigma }$. The final optimization problem in terms of $\varvec{\xi }_w$ is

19 $$\begin{aligned} \widehat{\varvec{\xi }}_{w} = & \mathop {{\text{argmin}}}\limits_{{\varvec{\xi }_{w} }} \Psi ({\mathbf{L}}\varvec{\xi }_{w} ) \\ \end{aligned}$$

20 $$ \begin{aligned}=  & \mathop {{\text{argmin}}}\limits_{{\varvec{\xi }_{w} }} \mathop \sum \limits_{i}^{N} \left[ {{\mathbf{A}}f^{{ - 1}} ({\mathbf{L}}\varvec{\xi }_{w} )} \right. - {\mathbf{y}} \odot \log ({\mathbf{A}}f^{{ - 1}} ({\mathbf{L}}\varvec{\xi }_{w} ))]_{i}  + \frac{1}{2}\varvec{\xi }_{w}^{\textsf{T}} \varvec{\xi }_{w} ,\\  \end{aligned}  $$

where $\odot$ is the element-wise product operation. Note that (19) is an unconstrained optimization problem, and it can be solved with a general purpose global optimization algorithms. We find that quasi-Newton algorithms, such as the Limitied memory Broyden-Fletcher-Goldfarb-Shanno (L-BFGS)^44^ achieves swift convergence. The gradient of (19), which is required for many optimization algorithms, can be analytically found as follows:

21 $$\nabla _{{\varvec{\xi }_{w} }} \Psi ({\mathbf{L}}\varvec{\xi }_{w} ) = {\mathbf{L}}^{\textsf{T}} \left(\left( {{\mathbf{A}}^{\textsf{T}} - {\mathbf{A}}^{\textsf{T}}\mathbb {1} \left( {{\mathbf{y}} {\oslash }\left( {{\mathbf{A}}\varvec{x}} \right)} \right)} \right) \odot \left(\mathbb {1}{\oslash }\left( {\sqrt {2\pi } \lambda {\text{diag}}({\mathbf{\Sigma }})} \right) \odot \exp \left( {\lambda \varvec{x} - \varvec{\xi }^{{ \circ 2}} {\oslash }\left( {2{\text{diag}}({\mathbf{\Sigma }})} \right)} \right)\right)\right) + \varvec{\xi }_{w} ,$$

where $^{\circ }$ is the element-wise power operations. Also, the $\text{diag}{(\cdot )}$ operator extracts the main diagonal elements of a matrix into a vector. The derivation of the gradient expression (21) can be found in the "Supplementary information". Once the optimization problem is solved and $\hat{\varvec{\xi }}_w$ is obtained, the image $\hat{\textbf{x}}$ can be obtained by $\hat{\textbf{x}}=\varvec{f}^{-1}(\textbf{L}\hat{\varvec{\xi }}_w)$. The pseudocode for the GPP MAP estimation is shown in Algorithm 1.

**Algorithm 1:**
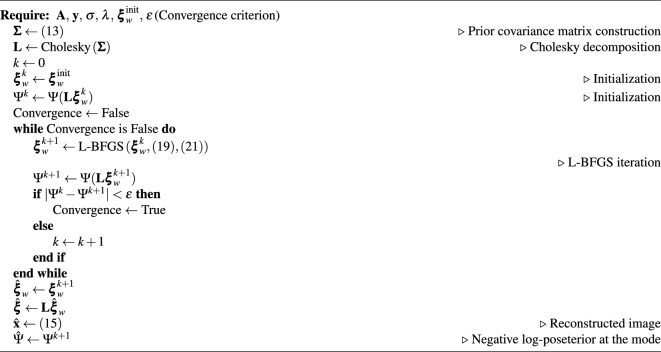
GPP MAP estimation

#### Kronecker product representation of the covariance matrix

With a large number of voxels *N*, setting up and solving (19) is a burdensome task due mainly to the computational costs involved in the Cholesky factor $\textbf{L} \in \mathbb {R}^{N \times N}$. For example, storing and computing the Cholesky factor requires $\mathscr {O}(N^2)$ memory and $\mathscr {O}(N^3)$ time, which can be prohibitively expensive for a large *N*.

Computation of the Cholesky decomposition is also the main computational bottleneck in the GP regression problems. To overcome the challenge, in^45^ Saatci et al. showed that when the GP regression input variables lie on a multi-dimensional Cartesian grid, the Kronecker structure in the covariance matrix $\varvec{\Sigma }$ can be exploited to significantly lessen the computational costs involving the Cholesky factor $\textbf{L}$. As many imaging problems employ Cartesian grid imaging spaces, here the strategy can be readily extended to the GPP image reconstruction.

When a covariance kernel function is the product of kernels across each Cartesian input dimension, such that $k(\textbf{r}_i, \textbf{r}_j) = \prod _{d=1}^{D}k({r_i^d}, {r_j^d})$, the covariance matrix $\varvec{\Sigma }$ constructed for a multi-dimensional lattice imaging space can be expressed as a Kronecker product of much smaller covariance matrices for each input dimension $\varvec{\Sigma }_d$ (i.e., *x*-, *y*- and *z*-dimensions), such that $\varvec{\Sigma } = \bigotimes _{d=1}^D \varvec{\Sigma }_d$. Furthermore, the Cholesky factor of a such covariance matrix is also a Kronecker product of Cholesky factor in each dimension, $\textbf{L} = \bigotimes _{d=1}^D \textbf{L}_d$. Hence, assuming the same number of voxels in each dimension, the memory and time complexity of the Cholesky factor computation are eased with $\mathscr {O}(DN^{2/D})$ and $\mathscr {O}(DN^{3/D})$.

Even with the obtained Kronecker product of the Cholesky factor, a naive matrix-vector multiplication $\textbf{L}\varvec{\xi }_w$ in (19) and (21) limits the scalability of the algorithm. Surprisingly, multiplication of a Kronecker product matrix on a vector can be cast as a tensor outer product and performed in $\mathscr {O}(DN^\frac{1+D}{D})$ time^45^^46^.

Equipped with the Kronecker product covariance matrix and its Cholesky factor, the overall time complexity of computing the cost function (19) and its gradient (21) is determined by the forward projection $\textbf{A}f^{-1}(\textbf{L}\varvec{\xi }_w)$, which is computed in $\mathscr {O}(MN)$. Note that the ML-EM has the same complexity of $\mathscr {O}(MN)$ per iteration.

#### Incorporation of structural prior

With the growing interest in contextual data fusion and multi-modality radiation imaging^47–49^, we discuss here the incorporation of a priori structural information using the GPP. Such a priori structural information can be obtained from other radiation imaging modalities (e.g., MRI, CT), commercial sensors (e.g., LiDAR, visual camera), or object detection algorithms.

We consider an imaging space $\mathscr {X}$ with *N* voxels $\mathscr {X}=\{x_1, x_2, \dots x_N\}$ and further, *L* voxel clusters $C_l \subseteq \mathscr {X}$ for $l=1, 2, \dots L$, each belonging to different objects or structures in the imaging space $\mathscr {X}$. This structural information can be incorporated by modifying the covariance matrix of the Gaussian prior $\varvec{\Sigma }$ (11). The modified covariance matrix is given by:

22 $$\begin{aligned} \Sigma _{ij} = {\left\{ \begin{array}{ll} k(\textbf{r}_i, \textbf{r}_j),& \text {if } x_i, x_j \in C_l \quad \forall l=1,2,\dots ,L \\ 0, & \text {otherwise}. \end{array}\right. } \end{aligned}$$

In other words, spatial correlation is defined only within the same object $C_l$. Note that different hyperparameters $\sigma$ and $\lambda$ can be applied to each voxel cluster $C_l$. The benefit of incorporating a structural prior is that it introduces correlations within salient features in the imaging space, leading to improved image quality and interpretability of the reconstruction results.

Note that, with the use of structural prior, the covariance matrix cannot be represented as a Kronecker product; however, by assigning zeroes for the covariances between different voxel clusters, the covariance matrix becomes a sparse block diagonal matrix, whose Cholesky decomposition can be computed and stored efficiently.

### Bayesian uncertainty quantification

The use of a Gaussian process prior in this work furnishes the basis for further Bayesian inference such as Bayesian uncertainty quantification. In Bayesian statistics, the uncertainty in the posterior distribution $p(\textbf{x}|\textbf{y})$ is often expressed in terms of a Bayesian credible interval. For a marginalized posterior variable $x_j$, the $(1-\alpha )$100% Bayesian credible interval is defined as the range $[x_{j-}, x_{j+}]$ within which $x_j$ falls with a probability of $1-\alpha$. In other words, $p(x_j \in [x_{j-}, x_{j+}]|\textbf{y}) = 1-\alpha$. The choice of such interval is not unique; therefore, we use an equal-tailed interval to define $x_{j-}$ and $x_{j+}$ as follows:

23 $$\begin{aligned}&\int _{-\infty }^{x_{j-}} p(x_j|\textbf{y}) \, dx_j = \frac{\alpha }{2} \end{aligned}$$

24 $$\begin{aligned}&\int _{x_{j+}}^{\infty } p(x_j|\textbf{y}) \, dx_j = \frac{\alpha }{2}. \end{aligned}$$

However, computation of the credible intervals is a daunting task, as it requires the integration of a very high dimensional distribution, $p(\textbf{y}) = \int p(\textbf{y}|\varvec{x})p(\varvec{x})\,d\varvec{x}$ (i.e., the marginal probability). Therefore, in this work, we investigate two different posterior approximation methods for credible interval estimation.

#### Preconditioned Crank–Nicolson (pCN) algorithm

In Bayesian statistics, Markov chain Monte Carlo (MCMC) algorithms have been widely used to generate a sequence of samples from an analytically intractable posterior distribution. However, for high dimensional posterior distributions (e.g., images), the MCMC sample convergence rate becomes very slow^50^.

In our approach, the use of GP prior in (19) lends itself to the use of the pCN algorithm^15^, a dimension-robust MCMC algorithm specifically designed for posterior distributions with a Gaussian prior. The pCN convergence rate is independent of discretization of a parameter space, which is an attractive feature for high-dimensional inverse problems such as image reconstruction.

With the pCN algorithm, a chain of *J* samples $\{\varvec{\xi }_n\}_{n=1}^J$ is obtained by following the proposal and acceptance/rejection steps. First, the pCN proposal in terms of $\varvec{\xi }$ is given by,

25 $$\begin{aligned} \varvec{\xi }^{\prime }_{n+1} = \sqrt{1-\beta ^2}\varvec{\xi }_{n} + \beta \varvec{\Xi }_{n+1}, \end{aligned}$$

where $\varvec{\Xi }_{n+1}$ is a sample from $\mathscr {N}(\textbf{0},\varvec{\Sigma })$ and the hyperparameter $0<\beta <1$ is a step size which is chosen manually. With the obtained $\varvec{\xi }^{\prime }_{n+1}$, the acceptance probability is computed as

26 $$\begin{aligned} \alpha (\varvec{\xi }_{n},\ \varvec{\xi }^{\prime }_{n+1}) = \min \, (1,\,\exp {(l(\varvec{\xi }_n;\textbf{y})-l(\varvec{\xi }_{n+1};\textbf{y}))}). \end{aligned}$$

Subsequently, the acceptance probability $\alpha (\varvec{\xi }_{n},\ \varvec{\xi }^{\prime }_{n+1})$ is compared to a random number $Z_{n+1}$ drawn from a uniform distribution $Z_{n+1}\sim {\text {Unif}}([0,1])$ and sets

27 $$\begin{aligned} \varvec{\xi }_{n+1} = {\left\{ \begin{array}{ll} \varvec{\xi }^{\prime }_{n+1}, & \text {if } Z_{n+1}<\alpha (\varvec{\xi }_{n},\ \varvec{\xi }^{\prime }_{n+1}) \\ \varvec{\xi }_{n}, & \text {otherwise.} \end{array}\right. } \end{aligned}$$

In our experiments, the MCMC sample chains were started from the reconstructed image (i.e., the mode of the posterior). Once a sufficient number of samples are obtained, the pixelwise $(1-\alpha )100$% confidence intervals can be found as follows. First, compute the pixelwise $({\alpha }/{2})100\%$ and $(1-{\alpha }/{2})100\%$ quantiles $\varvec{\xi }_{-}$ and $\varvec{\xi }_{+}$ from the obtained samples $\{\varvec{\xi }_n\}_{n=1}^J$. Then, the quantiles in $\textbf{x}$ are obtain as $\textbf{x}_{-}=\varvec{f}^{-1}(\varvec{\xi }_{-})$ and $\textbf{x}_{+}=\varvec{f}^{-1}(\varvec{\xi }_{+})$. As the inverse link function $\varvec{f}^{-1}$ is monotonically increasing, it ensures that the quantiles of $\textbf{x}$ can be directly found by transforming the quantiles of $\varvec{\xi }$. Lastly, the pixelwise $(1-\alpha )100$% credible interval map $\textbf{x}_\text {CI}$ can be found as $\textbf{x}_\text {CI}=\textbf{x}_{+}-\textbf{x}_{-}$.

Collecting *S* samples, the time complexity of the pCN method is $\mathscr {O}(MNS)$. Although each sample can be taken efficiently, ensuring convergence of the MCMC chain requires a large *S*, on the order of $10^5$ to $10^6$ samples. In the next section, we explore an alternative approach to posterior approximation, which typically runs much faster than the pCN method.

#### Laplace approximation

Another posterior estimation method, the Laplace approximation, uses a multivariate Gaussian distribution with the mean located at the MAP estimate $\hat{\varvec{\xi }}$ found from (19) to directly approximate a posterior distribution.

Using second-order Taylor expansion of the unnormalized log posterior $-\Psi (\varvec{\xi })$ at the mode $\hat{\varvec{\xi }}$, the posterior distribution can be approximated with $q(\varvec{\xi }|\varvec{y})$ as follows:

28 $$\begin{aligned}&p(\varvec{\xi }|\varvec{y}) \approx q(\varvec{\xi }|\varvec{y}), \quad \text {where} \end{aligned}$$

29 $$\begin{aligned}&q(\varvec{\xi }|\varvec{y}) \sim \mathscr {N}\left( \hat{\varvec{\xi }}, \left( \nabla ^2_{\varvec{\xi }}\Psi ({\varvec{\xi }})|_{\varvec{\xi }=\hat{\varvec{\xi }}}\right) ^{-1} = \left( \nabla ^2_{\varvec{\xi }} l(\varvec{\xi };\varvec{y})|_{\varvec{\xi }=\hat{\varvec{\xi }}} + \varvec{\Sigma }^{-1}\right) ^{-1}\right) , \end{aligned}$$

A more detailed derivation as well as the expression for $\nabla ^2_{\varvec{\xi }}\Psi ({\varvec{\xi }})|_{\varvec{\xi }=\hat{\varvec{\xi }}}$ is presented in the "Supplementary information". Once the approximate posterior distribution $q(\varvec{\xi }|\textbf{y})$ is obtained, the pixelwise $({\alpha }/{2})100\%$ and $(1-{\alpha }/{2})100\%$ quantiles $\varvec{\xi }_{-}$ and $\varvec{\xi }_{+}$ are found directly from the Gaussian distribution quantile functions. Then, the $(1-\alpha )100$% pixelwise credible interval map $\textbf{x}_\text {CI}$ can be obtained as $\textbf{x}_\text {CI} = \varvec{f}^{-1}(\varvec{\xi }_{+}) - \varvec{f}^{-1}(\varvec{\xi }_{-})$. Note that the matrix $\nabla ^2_{\varvec{\xi }}\Psi ({\varvec{\xi }})|_{\varvec{\xi }=\hat{\varvec{\xi }}}$ has the dimension of $N \times N$. With moderate number of voxles (i.e., N$<10^4$), the inversion in (29) can be directly performed. However, with a large number of voxels, instantiating the covariance matrix and computing the inversion in (29) can be computationally very expensive.

In order for more efficient covariance matrix computation, the Hessian of the log-likelihood can be approximated with the Fisher information matrix at the mode $\textbf{F}(\hat{\varvec{\xi }})$,

30 $$\begin{aligned} \nabla ^2_{\varvec{\xi }} l(\varvec{\xi };\varvec{y})|_{\varvec{\xi }=\hat{\varvec{\xi }}}&\approx \mathbb {E}_{\textbf{y} \sim p(\textbf{y}|\bar{\textbf{y}})}\left[ \left( \nabla _{\varvec{\xi }} l(\varvec{\xi };\varvec{y})|_{\varvec{\xi }=\hat{\varvec{\xi }}}\right) \left( \nabla _{\varvec{\xi }} l(\varvec{\xi };\varvec{y})|_{\varvec{\xi }=\hat{\varvec{\xi }}}\right) ^ \textsf{T} \right] \end{aligned}$$

31 $$\begin{aligned}&= (\textbf{J}_{\varvec{\xi }}{\varvec{f}^{-1}(\varvec{\xi })}|_{\varvec{\xi }=\hat{\varvec{\xi }}})^\textsf{T} \textbf{A}^{\textsf{T}} \text{diag}\left( (\textbf{A}\varvec{f}^{-1}(\varvec{\xi }))^{\circ -1}\right) \textbf{A}(\textbf{J}_{\varvec{\xi }}{\varvec{f}^{-1}(\varvec{\xi })}|_{\varvec{\xi }=\hat{\varvec{\xi }}}) \end{aligned}$$

32 $$\begin{aligned}&= \textbf{BB}^\textsf{T}, \end{aligned}$$

where we define $\textbf{B} \equiv (\textbf{J}_{\varvec{\xi }}{\varvec{f}^{-1}(\varvec{\xi })}|_{\varvec{\xi }=\hat{\varvec{\xi }}})^\textsf{T} \textbf{A}^{\textsf{T}} \text{diag}\left( (\textbf{A}\varvec{f}^{-1}(\varvec{\xi }))^{\circ -\frac{1}{2}}\right)$. The detailed derivation of (31) and the expression for $\textbf{J}_{\varvec{\xi }}{\varvec{f}^{-1}(\varvec{\xi })}$ is presented in the accompanying "Supplementary information". Fisher information has been studied extensively for neural network Hessian approximation, and it is well-justified as it is the expected Hessian of log-likelihood under $p(\textbf{y}|\bar{\textbf{y}})$. Equipped with the Hessian of the log-likelihood approximation, the expression for the covariance matrix in (29) becomes

33 $$\begin{aligned} \left( \nabla ^2_{\varvec{\xi }}\Psi ({\varvec{\xi }})|_{\varvec{\xi }=\hat{\varvec{\xi }}}\right) ^{-1}&\approx (\textbf{B}\textbf{B}^\textsf{T} + \varvec{\Sigma }^{-1})^{-1} \end{aligned}$$

34 $$\begin{aligned}&= \textbf{L}(\textbf{L}^{\textsf{T}}\textbf{B}\textbf{B}^\textsf{T}\textbf{L} + \textbf{I})^{-1}\textbf{L}^{\textsf{T}}. \end{aligned}$$

Next, we perform rank-K truncated eigendecomposition on $\textbf{L}^{\textsf{T}}\textbf{B}\textbf{B}^\textsf{T}\textbf{L}$, such that $\textbf{L}^{\textsf{T}}\textbf{B}\textbf{B}^\textsf{T}\textbf{L} \approx \textbf{Q}\textbf{S}\textbf{Q}^{\textsf{T}}$ where $\textbf{Q} \in \mathbb {R}^{N \times K}$ is a matrix containing eigenvectors and $\textbf{S} \in \mathbb {R}^{K \times K}$ is a diagonal matrix whose elements contain *K* largest eigenvalues of $\textbf{L}^{\textsf{T}}\textbf{B}\textbf{B}^\textsf{T}\textbf{L}$. Such decomposition can be efficiently obtained from randomized eigendecomposition algorithms. Randomized decomposition methods first proceed by forming a matrix $\textbf{Y} \in \mathbb {R}^{N \times K}$ by multiplying a random matrix $\textbf{P} \in \mathbb {R}^{N \times K}$ such that

35 $$\begin{aligned} \textbf{Y} = (\textbf{L}^{\textsf{T}}\textbf{B}(\textbf{B}^\textsf{T}\textbf{L}\textbf{P})). \end{aligned}$$

The matrix $\textbf{Y}$ captures most of the column space in $\textbf{L}^{\textsf{T}}\textbf{B}\textbf{B}^\textsf{T}\textbf{L}$ yet considerably smaller with $K \ll \text {min}(M, N)$. Therefore, it can be further processed to efficiently estimate the eigendecomposition of $\textbf{L}^{\textsf{T}}\textbf{B}\textbf{B}^\textsf{T}\textbf{L}$. More details on the randomized eigendecomposition methods can be found in Halko et al.^51^. When the system matrix $\textbf{A}$ is computed on-the-fly or too large to be loaded into core memory, the decomposition can be obtained efficiently with only single-pass of $\textbf{A}$ using the matrix sketch algorithm^52^. Also, note that by computing the matrix-matrix multiplication in the order specified by the parentheses in (35), we never instantiate an $N \times N$ matrix, which is potentially too large to be stored in memory.

Once the eigendecomposition is obtained, the covariance matrix can be computed as follows:

36 $$\begin{aligned} \left( \nabla ^2_{\varvec{\xi }}\Psi ({\varvec{\xi }})|_{\varvec{\xi }=\hat{\varvec{\xi }}}\right) ^{-1}&\approx \textbf{L}(\textbf{Q}\textbf{S}\textbf{Q}^{\textsf{T}} + \textbf{I})^{-1}\textbf{L}^{\textsf{T}} \end{aligned}$$

37 $$\begin{aligned}&=\textbf{L}(\textbf{I}-\textbf{Q}(\textbf{S}^{-1} + \textbf{Q}^{\textsf{T}}\textbf{Q})^{-1}\textbf{Q}^{\textsf{T}})\textbf{L}^\textsf{T}\end{aligned}$$

38 $$\begin{aligned}&=\textbf{L}(\textbf{I}-\textbf{Q}(\textbf{S} \oslash (\textbf{S} + \textbf{I}))\textbf{Q}^{\textsf{T}})\textbf{L}^\textsf{T}\end{aligned}$$

39 $$\begin{aligned}&=\textbf{L}\textbf{L}^\textsf{T}-\textbf{L}\textbf{Q}(\textbf{S} \oslash (\textbf{S} + \textbf{I}))\textbf{Q}^{\textsf{T}}\textbf{L}^\textsf{T} \end{aligned}$$

The second equality follows from the Woodbury matrix identity^53^. The resulting expression (39) for the covariance matrix can be partially computed to obtain necessary covariance elements for uncertainty quantification. For example, to create a pixel-wise uncertainty map, only the diagonal elements can be computed without forming an $N \times N$ matrix.

Due to the unimodality of the approximating Gaussian distribution, the Laplace approximation is well-justified when the posterior density is concentrated around the mode of the posterior, $\hat{\varvec{\xi }}$. The so-called “Bayesian central limit theorem” (i.e., Berstein-von Mises theorem) states that with increasing number of conditionally independent measurements, posterior distributions tend toward Gaussian distributions under certain conditions^54^. Hence, with a sufficient number of measurements, the Laplace approximation provides fast, yet reasonably accurate approximation of the posterior distribution.

### Hyperparameter selection with empirical Bayes

Although the hyperparameters of the GPP offers good physical and statistical interpretability, a principled method to infer the hyperparamters from the measurement is highly desirable. Here we show that the hyperparameters $\sigma$ and $\lambda$ can be systematically determined with the empirical Bayes method.

Including the hyperparameter vector $\varvec{\theta }=[\sigma , \lambda ]^\textsf{T}$, the Bayes rule (8) can be re-written in terms of $\varvec{\xi }$ as

40 $$\begin{aligned} p(\varvec{\xi }|\textbf{y}, \varvec{\theta }) =&\,\frac{p(\textbf{y}|\varvec{\xi }, \varvec{\theta })p(\varvec{\xi } | \varvec{\theta })}{p(\textbf{y} | \varvec{\theta })},\quad \text {where} \end{aligned}$$

41 $$\begin{aligned} p(\textbf{y} | \varvec{\theta }) =&\,\int p(\textbf{y}|\varvec{\xi }, \varvec{\theta })p(\varvec{\xi } | \varvec{\theta })\,d\varvec{\xi }. \end{aligned}$$

The empirical Bayes approach finds the hyperparameters $\hat{\varvec{\theta }}$ maximizing the marginal likelihood as follows:

42 $$\begin{aligned} \hat{\varvec{\theta }} = \mathop {\text{argmin}}\limits _{\varvec{\theta }, \varvec{\theta } \succ 0} -\log {p(\textbf{y} | \varvec{\theta })} = \mathop {\text{argmin}}\limits _{\varvec{\theta }, \varvec{\theta } \succ 0} \Phi (\varvec{\theta }), \end{aligned}$$

where $\Phi (\varvec{\theta }) {\mathop {=}\limits ^{c}} -\log {p(\textbf{y} | \varvec{\theta })}$ is the unnormalized log-marginal likelihood. However, as the computation of $\Phi (\varvec{\theta })$ is intractable due to the high dimensional integration in (40), we use the Laplace approximation to estimate $\Phi (\varvec{\theta })$^55^. With the Laplace approximation, the unnormalized negative log-marginal likelihood $\Phi (\varvec{\theta })$ at the posterior mode $\hat{\varvec{\xi }}$ is given as

43 $$\begin{aligned} \Phi (\varvec{\theta }) \approx {\frac{1}{2}}\log {|\textbf{I}+\nabla ^2_{\varvec{\xi }} l(\varvec{\xi };\varvec{y})|_{\varvec{\xi }=\hat{\varvec{\xi }}}\varvec{\Sigma }|} + \Psi (\hat{\varvec{\xi }}). \end{aligned}$$

The derivation can be found in the "Supplementary information". Note that in (43), the $\Psi (\hat{\varvec{\xi }})$ term is an output of Algorithm 1. The computation of the log-determinant term has the complexity of $\mathscr {O}(N^3)$, which prevents scalable computation of the log-marginal likelihood when *N* is large. Therefore, we exploit the low-rank structure of the matrix $\nabla _{\varvec{\xi }} l(\varvec{\xi };\varvec{y})|_{\varvec{\xi }=\hat{\varvec{\xi }}}\varvec{\Sigma }$ to accelerate the computation of the log determinant.

As in (31), using the Fisher information matrix approximation to $\nabla ^2_{\varvec{\xi }} l(\varvec{\xi };\varvec{y})|_{\varvec{\xi }=\hat{\varvec{\xi }}}$, the determinant can be approximated as,

44 $$\begin{aligned} \Phi (\varvec{\theta }) \approx {\frac{1}{2}}\log {|\textbf{I}+\textbf{BB}^\textsf{T}\varvec{\Sigma }|} + \Psi (\hat{\varvec{\xi }}). \end{aligned}$$

We also observe that using the precomputed Cholesky decomposition $\varvec{\Sigma }=\textbf{LL}^\textsf{T}$, the matrix determinant lemma enables the following reformulation of the log-determinant term,

45 $$\begin{aligned} {\frac{1}{2}}\log {|\textbf{I}+\textbf{BB}^\textsf{T}\varvec{\Sigma }|} =&\, {\frac{1}{2}}\log {|\textbf{I}+\textbf{B}^\textsf{T}\textbf{LL}^\textsf{T}\textbf{B}|} \end{aligned}$$

46 $$\begin{aligned} =&\, {\frac{1}{2}}\log {|\textbf{I}+\textbf{L}^\textsf{T}\textbf{BB}^\textsf{T}\textbf{L}|}. \end{aligned}$$

The Kronecker structure of the Cholesky factor $\textbf{L}$ allows for very efficient matrix-matrix multiplication $\textbf{B}^\textsf{T}\textbf{L}$ or $\textbf{L}^\textsf{T}\textbf{B}$ in (45) and (46). Furthermore, depending on the dimensions of the system matrix $\textbf{A}$, (45) (when $M \ll N$) or (46) (when $N \ll M$) can be used to compute the log determinant efficiently.

**Algorithm 2:**
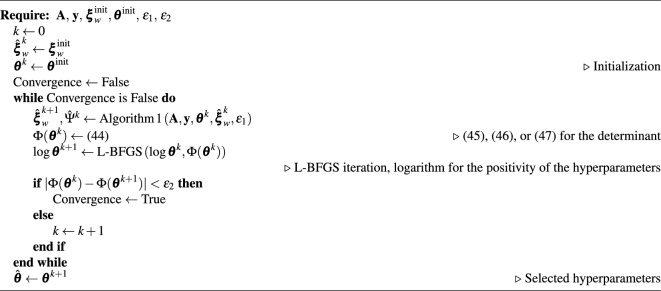
Hyperparameter selection with empirical Bayes

When both the *M* and *N* are too large, a low rank approximation of the system matrix $\textbf{A} \approx \textbf{U}_K\textbf{V}_K^{\textsf{T}}$ where $\textbf{U}_K \in \mathbb {R}^{M \times K}$ and $\textbf{V}_K \in \mathbb {R}^{N \times K}$ with $K \ll M, N$ can be used to accelerate the computation. Defining $\textbf{C} = \textbf{V}_K^{\textsf{T}}\textbf{J}_{\varvec{\xi }}{\varvec{f}^{-1}(\varvec{\xi })}|_{\varvec{\xi } = \hat{\varvec{\xi }}}\textbf{L}$ and $\textbf{D} = \textbf{U}_K^{\textsf{T}} {\text {diag}}{(\mathbb {1}\oslash \bar{\textbf{y}}^{\circ \frac{1}{2}})}$, the log-determinant term can be approximated as,

47 $$\begin{aligned} {\frac{1}{2}}\log {|\textbf{I}+\textbf{W}\varvec{\Sigma }|} \approx {\frac{1}{2}}\log {|\textbf{I}+\textbf{CC}^\textsf{T}\textbf{DD}^\textsf{T}|}. \end{aligned}$$

Hence using the rank-*K* approximation, the log-determinant computation has $\mathscr {O}(K^3)$ time complexity. The low-rank approximation of $\textbf{A}$ needs to be computed only once using various techniques, and we found that randomized singular value decomposition (rSVD) provides a good approximation with the time complexity of $\mathscr {O}(MN\log {K}+(M+N)K^2)$^51^. Such approximation of $\textbf{A}$ may sacrifice some accuracy in image reconstruction; however, we found that the approximation has negligible impact for hyperparameter selection and once the hyperparameters are chosen, the full-rank system matrix can be used for more accurate image reconstruction. Additionally, the low rank approximation can also reduce the computation of forward projection $\textbf{A}\textbf{f}^{-1}(\varvec{\xi })$, which helps to accelerate the overall optimization process.

Once an adequate log-marginal likelihood computation scheme is selected, the L-BFGS can be used to solve the optimization problem (42). The necessary gradient of $\Phi (\varvec{\theta })$ with respect to $\varvec{\theta }$ can be found with finite difference methods. The entire marginal likelihood maximization scheme is illustrated in Algorithm 2.

## Supplementary Information

Supplementary Information.

## Data Availability

The datasets used and/or analysed during the current study available from the corresponding author on reasonable request.

## Code availability

The code used for analysis can be found at https://github.com/jwonl/GPP_public.
