# Supplementary material for: Radiation image reconstruction and uncertainty quantification using a Gaussian process prior
Source: Sci Rep. 2024 Oct 3;14:22958. doi: 10.1038/s41598-024-71336-z (PMC11452213; doi:10.1038/s41598-024-71336-z)
Supplement: Supplementary file 1 — Supplementary Information. [file 41598_2024_71336_MOESM1_ESM.pdf]

Supplementary Infomation for Radiation Image Reconstruction and Uncertainty Quantification Using a Gaussian Process Prior

Jaewon Lee[1*], Tenzing H. Joshi[2], Mark S. Bandstra[2], Donald L. Gunter[3], Brian J. Quiter[2], Reynold J. Cooper[2], and Kai Vetter[1,2]

[1]Department of Nuclear Engineering, University of California, Berkeley, Berkeley, 94720 CA, USA
[2]Applied Nuclear Physics (ANP) Program, Lawrence Berkeley National Laboratory, Berkeley, 94720 CA, USA
[3]Gunter Physics, Inc., Lisle, 60532 IL, USA
[*]jwonlee@berkeley.edu

Supplementary Information

Derivation of the GPP Posterior Gradient

The gradient of the negative log-posterior $\Psi(\mathbf{L}\boldsymbol{\xi}_w)$ can be obtained as follows. Adopting the convention that the gradient of a scalar function is a column vector,

$$\nabla_{\boldsymbol{\xi}_w}\Psi(\mathbf{L}\boldsymbol{\xi}_w) \tag{1}$$

$$\begin{aligned} &= \nabla_{\boldsymbol{\xi}_w} l(\mathbf{L}\boldsymbol{\xi}_w;\mathbf{y}) + \nabla_{\boldsymbol{\xi}_w} l_p(\mathbf{L}\boldsymbol{\xi}_w) \\ &= \mathbf{J}_{\boldsymbol{\xi}_w}\boldsymbol{\xi}^\top \mathbf{J}_{\boldsymbol{\xi}}\boldsymbol{f}^{-1}(\boldsymbol{\xi})^\top \nabla_{\boldsymbol{f}^{-1}(\boldsymbol{\xi})} l(\mathbf{L}\boldsymbol{\xi}_w;\mathbf{y}) + \boldsymbol{\xi}_w, \end{aligned} \tag{2}$$

where $\boldsymbol{x} = \boldsymbol{f}^{-1}(\mathbf{L}\boldsymbol{\xi}_w)$ and $\boldsymbol{\xi} = \mathbf{L}\boldsymbol{\xi}_w$. The second equality follws from the chain rule. Each of the gradients and Jacobians in (2) is given as

$$\nabla_{\boldsymbol{f}^{-1}(\boldsymbol{\xi})} l(\mathbf{L}\boldsymbol{\xi}_w;\mathbf{y}) = \mathbf{A}^\top\mathbb{1} - \mathbf{A}^\top(\mathbf{y}\oslash(\mathbf{A}\boldsymbol{x})), \tag{3}$$

$$\mathbf{J}_{\boldsymbol{\xi}}\boldsymbol{f}^{-1}(\boldsymbol{\xi}) = \operatorname{diag}\left(\mathbb{1}\oslash\left(\sqrt{2\pi}\lambda\operatorname{diag}(\boldsymbol{\Sigma})\right)\odot\exp\left(\lambda\boldsymbol{x} - \boldsymbol{\xi}^{\circ 2}\oslash(2\operatorname{diag}(\boldsymbol{\Sigma}))\right)\right), \tag{4}$$

$$\mathbf{J}_{\boldsymbol{\xi}_w}\boldsymbol{\xi} = \mathbf{L}. \tag{5}$$

Note that depending on the choice of a link function, the Jacobian $\mathbf{J}_{\boldsymbol{\xi}}\boldsymbol{f}^{-1}(\boldsymbol{\xi})$ is computed differently. The Jacobian given in (4) is for the exponential-to-Gaussian link function introduced in the main text. Combining (3), (4), and (5) together, the gradient is given as

$$\begin{aligned} &\nabla_{\boldsymbol{\xi}_w}\Psi(\mathbf{L}\boldsymbol{\xi}_w) = \\ &\mathbf{L}^\top\Bigg(\left(\mathbf{A}^\top\mathbb{1} - \mathbf{A}^\top(\mathbf{y}\oslash(\mathbf{A}\boldsymbol{x}))\right)\odot\Bigg(\mathbb{1}\oslash\left(\sqrt{2\pi}\lambda\operatorname{diag}(\boldsymbol{\Sigma})\right) \\ &\qquad\odot\exp\left(\lambda\boldsymbol{x} - \boldsymbol{\xi}^{\circ 2}\oslash(2\operatorname{diag}(\boldsymbol{\Sigma}))\right)\Bigg)\Bigg) + \boldsymbol{\xi}_w. \end{aligned} \tag{6}$$

Approximate Posterior and the Marginal Likelihood Using the Laplace Approximation

First we write the log-posterior distribution as $\log p(\boldsymbol{\xi}|\mathbf{y}) = \log\frac{1}{Z} - \Psi(\boldsymbol{\xi})$, where Z is the normalization constant. Then, the second order Taylor expansion of $-\Psi(\boldsymbol{\xi})$ around the mode of the posterior $\hat{\boldsymbol{\xi}} = \mathbf{L}\hat{\boldsymbol{\xi}}_w$ is given as,

$$-\Psi(\boldsymbol{\xi}) \approx -\Psi(\hat{\boldsymbol{\xi}}) - \frac{1}{2}(\hat{\boldsymbol{\xi}} - \boldsymbol{\xi})^\top \nabla^2_{\boldsymbol{\xi}}\Psi(\boldsymbol{\xi})\Big|_{\boldsymbol{\xi}=\hat{\boldsymbol{\xi}}}(\hat{\boldsymbol{\xi}} - \boldsymbol{\xi}) \tag{7}$$

Note that the first order term is not included because the gradient at the mode $-\nabla_{\xi}\Psi(\boldsymbol{\xi})\big|_{\xi=\hat{\xi}}$ is zero. Using the Taylor expansion, the log-posterior distribution is written as,

$$\log p(\boldsymbol{\xi}|\mathbf{y}) \approx \log\frac{1}{Z} - \Psi(\hat{\boldsymbol{\xi}}) - \frac{1}{2}(\hat{\boldsymbol{\xi}}-\boldsymbol{\xi})^{\mathsf{T}}\,\nabla^2_{\xi}\Psi(\boldsymbol{\xi})\Big|_{\xi=\hat{\xi}}(\hat{\boldsymbol{\xi}}-\boldsymbol{\xi}) \tag{8}$$

Exponentiating both sides of (8) to recover the posterior $p(\boldsymbol{\xi}|\mathbf{y})$, we have

$$p(\boldsymbol{\xi}|\mathbf{y}) \approx \frac{1}{Z}\exp\left(-\Psi(\hat{\boldsymbol{\xi}})\right)\exp\left(-\frac{1}{2}(\hat{\boldsymbol{\xi}}-\boldsymbol{\xi})^{\mathsf{T}}\,\nabla^2_{\xi}\Psi(\boldsymbol{\xi})\Big|_{\xi=\hat{\xi}}(\hat{\boldsymbol{\xi}}-\boldsymbol{\xi})\right) \tag{9}$$

Since (8) is in the form of Gaussian distribution, it immediately follows that,

$$p(\boldsymbol{\xi}|\mathbf{y}) \approx q(\boldsymbol{\xi}|\mathbf{y}), \qquad \text{where} \tag{10}$$

$$q(\boldsymbol{\xi}|\mathbf{y}) \sim \mathcal{N}\left(\hat{\boldsymbol{\xi}}, \left(\nabla^2_{\xi}\Psi(\boldsymbol{\xi})\Big|_{\xi=\hat{\xi}}\right)^{-1}\right), \tag{11}$$

The Hessian matrix $\nabla^2_{\xi}\Psi(\boldsymbol{\xi})$ is given as,

$$\nabla^2_{\xi}\Psi(\boldsymbol{\xi}) = \nabla^2_{\xi} l(\boldsymbol{\xi};\mathbf{y}) + \nabla^2_{\xi} l_p(\boldsymbol{\xi}) \tag{12}$$

where, $\nabla^2_{\xi} l_p(\boldsymbol{\xi}) = \boldsymbol{\Sigma}^{-1}$. The Hessian of the negative log-likelihood function $\nabla^2_{\xi} l(\boldsymbol{\xi};\mathbf{y})$ is given as,

$$\nabla^2_{\xi} l(\boldsymbol{\xi};\mathbf{y}) = \mathbf{J}_{\xi}\boldsymbol{f}^{-1}(\boldsymbol{\xi})\nabla^2_{\boldsymbol{f}^{-1}(\xi)} l(\boldsymbol{\xi};\mathbf{y})\mathbf{J}_{\xi}\boldsymbol{f}^{-1}(\boldsymbol{\xi}) + \nabla_{\boldsymbol{f}^{-1}(\xi)} l(\boldsymbol{\xi};\mathbf{y})\nabla^2_{\xi}\boldsymbol{f}^{-1}(\boldsymbol{\xi}). \tag{13}$$

The expressions for $\mathbf{J}_{\xi}\boldsymbol{f}^{-1}(\boldsymbol{\xi})$ and $\nabla_{\boldsymbol{f}^{-1}(\xi)} l(\boldsymbol{\xi};\mathbf{y})$ are given in (4) and (3). $\nabla^2_{\boldsymbol{f}^{-1}(\xi)} l(\boldsymbol{\xi};\mathbf{y})$ and $\nabla_{\boldsymbol{f}^{-1}(\xi)} l(\boldsymbol{\xi};\mathbf{y})\nabla^2_{\xi}\boldsymbol{f}^{-1}(\boldsymbol{\xi})$ are given as,

$$\nabla^2_{\boldsymbol{f}^{-1}(\xi)} l(\boldsymbol{\xi};\mathbf{y}) = \mathbf{A}^{\mathsf{T}}\operatorname{diag}\left(\frac{\mathbf{y}}{\bar{\mathbf{y}}^{\circ 2}}\right)\mathbf{A} \qquad \text{and} \tag{14}$$

$$\nabla_{\boldsymbol{f}^{-1}(\xi)} l(\boldsymbol{\xi};\mathbf{y})\nabla^2_{\xi}\boldsymbol{f}^{-1}(\boldsymbol{\xi}) = \operatorname{diag}\left(\nabla_{\boldsymbol{f}^{-1}(\xi)} l(\boldsymbol{\xi};\mathbf{y})\right)\mathbf{J}_{\xi}\boldsymbol{f}^{-1}(\boldsymbol{\xi})\left(\lambda\mathbf{J}_{\xi}\boldsymbol{f}^{-1}(\boldsymbol{\xi}) - (\boldsymbol{\xi}\oslash\operatorname{diag}(\boldsymbol{\Sigma}))\right). \tag{15}$$

Note that $\nabla^2_{\xi}\boldsymbol{f}^{-1}(\boldsymbol{\xi})$ is a 3-D diagonal tensor, but the computation of $\nabla_{\boldsymbol{f}^{-1}(\xi)} l(\boldsymbol{\xi};\mathbf{y})\nabla^2_{\xi} f^{-1}(\boldsymbol{\xi})$ is equivalent to (15). Combining (4), (3), (14) and (15), the Hessian of the negative log-likelihood function (13) is computed.

Fisher information for Hessian approximation

The Fisher information matrix provides a well-justified approximation to the Hessian of the negative log-likelihood $\nabla^2_{\xi} l(\boldsymbol{\xi};\boldsymbol{y})$. The approximation can be expressed as

$$\nabla^2_{\xi} l(\boldsymbol{\xi};\boldsymbol{y}) \approx \mathbb{E}_{\mathbf{y}\sim p(\mathbf{y}|\bar{\mathbf{y}})}\left[\left(\nabla_{\xi} l(\boldsymbol{\xi};\boldsymbol{y})|_{\xi=\hat{\xi}}\right)\left(\nabla_{\xi} l(\boldsymbol{\xi};\boldsymbol{y})|_{\xi=\hat{\xi}}\right)^{\mathsf{T}}\right], \tag{16}$$

where the right hand side is the definition of the Fisher information matrix. Using the chain rule, the gradient $\nabla_{\xi} l(\boldsymbol{\xi};\boldsymbol{y})|_{\xi=\hat{\xi}}$ can be expressed as,

$$\nabla_{\xi} l(\boldsymbol{\xi};\boldsymbol{y})|_{\xi=\hat{\xi}} = (\mathbf{J}_{\xi}\boldsymbol{f}^{-1}(\boldsymbol{\xi}))^{\mathsf{T}}(\mathbf{J}_{\boldsymbol{f}^{-1}(\xi)}\log\bar{\mathbf{y}})^{\mathsf{T}}\nabla_{\log\bar{\mathbf{y}}} l(\boldsymbol{\xi};\boldsymbol{y})|_{\xi=\hat{\xi}}, \tag{17}$$

where $\bar{\mathbf{y}} = \mathbf{A}\boldsymbol{f}^{-1}(\boldsymbol{\xi})$ is forward projection. Plugging (17) into (16), we have

$$\begin{aligned}
\nabla^2_{\xi} l(\boldsymbol{\xi};\boldsymbol{y}) &\approx \mathbb{E}_{\mathbf{y}\sim p(\mathbf{y}|\bar{\mathbf{y}})}\left[(\mathbf{J}_{\xi}\boldsymbol{f}^{-1}(\boldsymbol{\xi}))^{\mathsf{T}}(\mathbf{J}_{\boldsymbol{f}^{-1}(\xi)}\log\bar{\mathbf{y}})^{\mathsf{T}}\nabla_{\log\bar{\mathbf{y}}} l(\boldsymbol{\xi};\boldsymbol{y})|_{\xi=\hat{\xi}}\nabla_{\log\bar{\mathbf{y}}} l(\boldsymbol{\xi};\boldsymbol{y})|^{\mathsf{T}}_{\xi=\hat{\xi}}(\mathbf{J}_{\boldsymbol{f}^{-1}(\xi)}\log\bar{\mathbf{y}})(\mathbf{J}_{\xi}\boldsymbol{f}^{-1}(\boldsymbol{\xi}))\right] && (18)\\
&= (\mathbf{J}_{\xi}\boldsymbol{f}^{-1}(\boldsymbol{\xi}))^{\mathsf{T}}(\mathbf{J}_{\boldsymbol{f}^{-1}(\xi)}\log\bar{\mathbf{y}})^{\mathsf{T}}\mathbb{E}_{\mathbf{y}\sim p(\mathbf{y}|\bar{\mathbf{y}})}\left[\nabla_{\log\bar{\mathbf{y}}} l(\boldsymbol{\xi};\boldsymbol{y})|_{\xi=\hat{\xi}}\nabla_{\log\bar{\mathbf{y}}} l(\boldsymbol{\xi};\boldsymbol{y})|^{\mathsf{T}}_{\xi=\hat{\xi}}\right](\mathbf{J}_{\boldsymbol{f}^{-1}(\xi)}\log\bar{\mathbf{y}})(\mathbf{J}_{\xi}\boldsymbol{f}^{-1}(\boldsymbol{\xi})) && (19)\\
&= (\mathbf{J}_{\xi}\boldsymbol{f}^{-1}(\boldsymbol{\xi}))^{\mathsf{T}}(\mathbf{J}_{\boldsymbol{f}^{-1}(\xi)}\log\bar{\mathbf{y}})^{\mathsf{T}}\mathbb{E}_{\mathbf{y}\sim p(\mathbf{y}|\bar{\mathbf{y}})}\left[\nabla^2_{\log\bar{\mathbf{y}}} l(\boldsymbol{\xi};\boldsymbol{y})|_{\xi=\hat{\xi}}\right](\mathbf{J}_{\boldsymbol{f}^{-1}(\xi)}\log\bar{\mathbf{y}})(\mathbf{J}_{\xi}\boldsymbol{f}^{-1}(\boldsymbol{\xi})). && (20)
\end{aligned}$$

The equivalence between the expectation of the outer product and the Hessian follows from the property of the Fisher information matrix (see,[1]) The Jacobians and the Hessian in (20) are given as,

$$(\mathbf{J}_{\boldsymbol{f}^{-1}(\boldsymbol{\xi})} \log \bar{\mathbf{y}}) = \operatorname{diag}(\bar{\mathbf{y}}^{\circ -1})\mathbf{A} \tag{21}$$

$$\nabla^2_{\log \bar{\mathbf{y}}} l(\boldsymbol{\xi};\boldsymbol{y})|_{\boldsymbol{\xi}=\hat{\boldsymbol{\xi}}} = \operatorname{diag}(\bar{\mathbf{y}}). \tag{22}$$

The Jacobian $(\mathbf{J}_{\boldsymbol{\xi}} \boldsymbol{f}^{-1}(\boldsymbol{\xi}))$ is given in (4) for the exponential-to-Gaussian link function, and it may differ depending on the choice of a link function. Plugging the Jacobians and Hessian in (20), we have

$$\nabla^2_{\boldsymbol{\xi}} l(\boldsymbol{\xi};\boldsymbol{y}) \approx (\mathbf{J}_{\boldsymbol{\xi}} \boldsymbol{f}^{-1}(\boldsymbol{\xi}))^\top \mathbf{A}^\top \operatorname{diag}(\bar{\mathbf{y}}^{\circ -1}) \mathbb{E}_{\mathbf{y} \sim p(\mathbf{y}|\bar{\mathbf{y}})} [\operatorname{diag}(\bar{\mathbf{y}})] \operatorname{diag}(\bar{\mathbf{y}}^{\circ -1}) \mathbf{A} (\mathbf{J}_{\boldsymbol{\xi}} \boldsymbol{f}^{-1}(\boldsymbol{\xi})) \tag{23}$$

$$= (\mathbf{J}_{\boldsymbol{\xi}} \boldsymbol{f}^{-1}(\boldsymbol{\xi}))^\top \mathbf{A}^\top \operatorname{diag}(\bar{\mathbf{y}}^{\circ -1}) \operatorname{diag}(\bar{\mathbf{y}}) \operatorname{diag}(\bar{\mathbf{y}}^{\circ -1}) \mathbf{A} (\mathbf{J}_{\boldsymbol{\xi}} \boldsymbol{f}^{-1}(\boldsymbol{\xi})) \tag{24}$$

$$= (\mathbf{J}_{\boldsymbol{\xi}} \boldsymbol{f}^{-1}(\boldsymbol{\xi}))^\top \mathbf{A}^\top \operatorname{diag}(\bar{\mathbf{y}}^{\circ -1}) \mathbf{A} (\mathbf{J}_{\boldsymbol{\xi}} \boldsymbol{f}^{-1}(\boldsymbol{\xi})) \tag{25}$$

Marginal likelihood computation using the Laplace approximation

The marginal likelihood $p(\mathbf{y}|\boldsymbol{\theta})$ can be expressed as,

$$p(\mathbf{y}|\boldsymbol{\theta}) = \int \exp\left(\log p(\mathbf{y}|\boldsymbol{\xi}) p(\boldsymbol{\xi}|\boldsymbol{\theta})\right) d\boldsymbol{\xi} \tag{26}$$

Similarly to (8), the $\log p(\mathbf{y}|\boldsymbol{\xi}) p(\boldsymbol{\xi}|\boldsymbol{\theta})$ can be approximated using the second-order Taylor expansion around the mode,

$$\log p(\mathbf{y}|\boldsymbol{\xi}) p(\boldsymbol{\xi}|\boldsymbol{\theta}) \approx$$

$$\log p(\mathbf{y}|\hat{\boldsymbol{\xi}}) p(\hat{\boldsymbol{\xi}}|\boldsymbol{\theta}) - \frac{1}{2} \left(\hat{\boldsymbol{\xi}} - \boldsymbol{\xi}\right)^\top \nabla^2_{\boldsymbol{\xi}} \Psi(\boldsymbol{\xi})\Big|_{\boldsymbol{\xi}=\hat{\boldsymbol{\xi}}} \left(\hat{\boldsymbol{\xi}} - \boldsymbol{\xi}\right) \tag{27}$$

Plugging (27) into (26), we have,

$$p(\mathbf{y}|\boldsymbol{\theta}) = p(\mathbf{y}|\hat{\boldsymbol{\xi}}) p(\hat{\boldsymbol{\xi}}|\boldsymbol{\theta})$$

$$\int \exp\left(-\frac{1}{2} \left(\hat{\boldsymbol{\xi}} - \boldsymbol{\xi}\right)^\top \nabla^2_{\boldsymbol{\xi}} \Psi(\boldsymbol{\xi})\Big|_{\boldsymbol{\xi}=\hat{\boldsymbol{\xi}}} \left(\hat{\boldsymbol{\xi}} - \boldsymbol{\xi}\right)\right) d\boldsymbol{\xi} \tag{28}$$

The integration in (28) can be analytically computed as $\left((2\pi)^N \left| \left(\nabla^2_{\boldsymbol{\xi}} \Psi(\boldsymbol{\xi})\Big|_{\boldsymbol{\xi}=\hat{\boldsymbol{\xi}}}\right)^{-1} \right|\right)^{\frac{1}{2}}$ (i.e., Gaussian integration). Hence, the negative log-marginal likelihood up to constant, $\Phi(\boldsymbol{\theta}) \stackrel{c}{=} -\log p(\mathbf{y}|\boldsymbol{\theta})$ is given as,

$$\Phi(\boldsymbol{\theta}) = \Psi(\hat{\boldsymbol{\xi}}) + \frac{1}{2} \log \left| \left(\nabla^2_{\boldsymbol{\xi}} \Psi(\boldsymbol{\xi})\Big|_{\boldsymbol{\xi}=\hat{\boldsymbol{\xi}}}\right) \right| + \log |\boldsymbol{\Sigma}| \tag{29}$$

$$= \Psi(\hat{\boldsymbol{\xi}}) + \frac{1}{2} \log \left| \left(\nabla^2_{\boldsymbol{\xi}} l(\boldsymbol{\xi};\mathbf{y})\Big|_{\boldsymbol{\xi}=\hat{\boldsymbol{\xi}}} + \boldsymbol{\Sigma}^{-1}\right) \right| + \log |\boldsymbol{\Sigma}| \tag{30}$$

$$= \Psi(\hat{\boldsymbol{\xi}}) + \frac{1}{2} \log \left| \mathbf{I} + \nabla^2_{\boldsymbol{\xi}} l(\boldsymbol{\xi};\mathbf{y})\Big|_{\boldsymbol{\xi}=\hat{\boldsymbol{\xi}}} \boldsymbol{\Sigma} \right|. \tag{31}$$

Note that the expression for $\nabla^2_{\boldsymbol{\xi}} l(\boldsymbol{\xi};\mathbf{y})$ is given in (13).

References

1. Kunstner, F., Hennig, P. & Balles, L. Limitations of the empirical Fisher approximation for natural gradient descent. In *Advances in Neural Information Processing Systems*, vol. 32 (Curran Associates, Inc., 2019).
